# Fluorescence microscopy-based sensitive method to quantify dopaminergic neurodegeneration in a *Drosophila* model of Parkinson’s disease

**DOI:** 10.3389/fnins.2023.1158858

**Published:** 2023-06-26

**Authors:** Mohamad Ayajuddin, Rahul Chaurasia, Abhik Das, Priyanka Modi, Limamanen Phom, Zevelou Koza, Sarat Chandra Yenisetti

**Affiliations:** ^1^*Drosophila* Neurobiology Laboratory, Department of Zoology, Nagaland University (Central), Lumami, Nagaland, India; ^2^Sao Chang Government College, Tuensang, Nagaland, India; ^3^Patkai Christian College (Autonomous), Dimapur, Nagaland, India

**Keywords:** dopamine, neurodegeneration, fluorescence intensity, tyrosine hydroxylase, *Drosophila*

## Abstract

Death of dopaminergic (DAergic) neurons in the *substantia nigra pars compacta* of the human brain is the characteristic pathological feature of Parkinson’s disease (PD). On exposure to neurotoxicants, *Drosophila* too exhibits mobility defects and diminished levels of brain dopamine. In the fly model of sporadic PD, our laboratory has demonstrated that there is no loss of DAergic neuronal number, however, a significant reduction in fluorescence intensity (FI) of secondary antibodies that target the primary antibody-anti-tyrosine hydroxylase (TH). Here, we present a sensitive, economical, and repeatable assay to characterize neurodegeneration based on the quantification of FI of the secondary antibody. As the intensity of fluorescence correlates with the amount of TH synthesis, its reduction under PD conditions denotes the depletion in the TH synthesis, suggesting DAergic neuronal dysfunction. Reduction in TH protein synthesis is further confirmed through Bio-Rad Stain-Free Western Blotting. Quantification of brain DA and its metabolites (DOPAC and HVA) using HPLC-ECD further demonstrated the depleted DA level and altered DA metabolism as evident from enhanced DA turnover rate. Together all these PD marker studies suggest that FI quantification is a refined and sensitive method to understand the early stages of DAergic neurodegeneration. FI quantification is performed using ZEN 2012 SP2, a licensed software from Carl Zeiss, Germany. This method will be of good use to biologists, as it with few modifications, can also be implemented to characterize the extent of degeneration of different cell types. Unlike the expensive and cumbersome confocal microscopy, the present method using fluorescence microscopy will be a feasible option for fund-constrained neurobiology laboratories in developing countries.

## Introduction

The first study on the α-synuclein-mediated *Drosophila* model of Parkinson’s disease (PD) demonstrated that misexpression of human α-synuclein causes a progressive age-dependent locomotor dysfunction and concurrent loss of dopaminergic (DAergic) neurons, similar to pathological and clinical manifestations of PD in humans (Feany and Bender, 2000). Since then, numerous laboratories have been using the fly model to examine the effects of gene mutations or over-expression in PD (Auluck et al., 2002; Pesah et al., 2005; Park et al., 2006; Botella et al., 2009; Navarro et al., 2014; Sur et al., 2018; Bordet et al., 2021; Maitra et al., 2021; Rai and Roy, 2022).

*Drosophila* models of PD recapitulate critical PD phenotype, i.e., loss of DAergic neurons (Feany and Bender, 2000; Auluck et al., 2002; Chen and Feany, 2005; Cooper et al., 2006; Trinh et al., 2008, 2010; Barone et al., 2011; Hernandez-Vargas et al., 2011; Sur et al., 2018; Maitra et al., 2021). However, some groups reported no loss in neuronal numbers (Pesah et al., 2004, 2005; Menzies et al., 2005; Meulener et al., 2005; Whitworth et al., 2005; Navarro et al., 2014; Ayajuddin et al., 2022). Neurotoxins such as rotenone (ROT) and paraquat (PQ) have been used to develop the *Drosophila* model of sporadic PD. In them, specific loss of DAergic neurons was found with different concentrations of toxins (Coulom and Birman, 2004; Chaudhuri et al., 2007; Wang et al., 2007; Maitra et al., 2021; Chaouhan et al., 2022) or no alteration in the number of neurons was also reported (Menzies et al., 2005; Meulener et al., 2005; Navarro et al., 2014; Ayajuddin et al., 2022). The tyrosine hydroxylase (TH) immunostaining and green-fluorescent protein (GFP) reporter-based techniques have typically been approached to measure DAergic neurons in the whole *Drosophila* brain. The reduction in fluorescence intensity (FI) of TH immunostaining or GFP signal has been referred to as “neuronal dysfunction” (Navarro et al., 2014).

In the present study, we employed the PQ-induced *Drosophila* model of PD that was developed in our laboratory (Phom et al., 2014) and demonstrated that in PD brain DAergic neuronal number remains unaffected but the FI of the secondary antibody that targets the primary anti-TH antibody (TH is a rate-limiting enzyme in the dopamine synthesis and marker protein for DAergic neurons) decreased as compared with control suggesting the neurodegeneration. This is further confirmed by quantifying the brain TH protein through western blotting and altered brain DA metabolism using HPLC-ECD.

Here, we describe a sensitive fluorescence microscopy-based assay, which is less expensive and user-friendly as compared to cumbersome confocal microscopy, to characterize DAergic neuronal dysfunction even in the absence of loss of neuronal cell body, which enables the researcher to follow the progress of neurodegeneration.

## Materials and methods

### Fly husbandry

The male Oregon K (OK) flies of the *D. melanogaster* were used in the present study (OK strain procured from National *Drosophila* Stock Center, Mysuru University, Mysuru, Karnataka, India). The flies were reared in a fly incubator at 22°C ± 2°C with a 12-h (Hr) light/dark cycle (Percival, United States). A culture media constituting sucrose, yeast, agar-agar, and propionic acid was used to feed the flies (Phom et al., 2014). The flies were collected by mildly anesthetizing them with a few drops of diethyl ether. Each vial with fresh culture media contained not more than 25 flies. Every 3rd day, the collected flies were moved to a fresh media vial. The early health phase (4–5 days old) flies were used in this experiment.

### Chemicals

The required chemicals *viz.*, Sucrose (SRL, Maharashtra, India, Cat: 84973), Methyl viologen dichloride hydrate /Paraquat (PQ; Sigma-Aldrich, St. Louis, MO, United States, Cat: 856177) were used for feeding procedures. Phosphate buffered Saline (PBS; HiMedia, Maharashtra, India, Cat: ML023), Paraformaldehyde (Sigma-Aldrich, St. Louis, MO, United States, Cat: I58127), Triton X-100 (TX-100, Sigma-Aldrich, St. Louis, MO, United States, Cat: T8787), Normal Goat Serum (NGS; Vector Labs, CA, United States, Cat: S1000), VECTASHIELD® mounting medium (Vector Labs, CA, United States, Cat: H1000), Rabbit anti-Tyrosine hydroxylase (anti-TH) polyclonal primary antibody (Millipore, MA, United States, Cat: Ab152), and Goat anti-rabbit IgG H&L (TRITC labeled) polyclonal secondary antibody (Abcam, MA, United States, Cat: Ab6718) were used for immunostaining. NGS (Sigma-Aldrich, St. Louis, MO, United States, Cat: 2153), Protease inhibitor cocktail tablets (Sigma-Aldrich, St. Louis, MO, United States, Cat:S8830), RCDC assay reagent (Bio-Rad, CA, United States, Cat: 500-0120), TGX stain-free fast cast acrylamide (Bio-Rad, CA, United States, Cat:161-0183TA), Pre-stained plus dual color protein standard (Bio-Rad, CA, United States, Cat:161-0374), PVDF membrane (Bio-Rad, CA, United States, Cat: 162-0174), Goat anti-rabbit HRP antibody (Abcam, MA, United States, Cat: 205718), Clarity western ECL substrate (Bio-Rad, CA, United States, Cat: 170-5060) were used in western blotting. Trichloro Acetic Acid (TCA; SRL, Maharashtra, India, Cat: 204842), Dopamine (DA; Sigma-Aldrich, St. Louis, MO, United States, Cat: H8502); 3,4-Dihydroxyphenylacetic acid (DOPAC; Sigma-Aldrich, St. Louis, MO, United States, Cat: 11569); Homo vanillic acid (HVA; Sigma-Aldrich, St. Louis, MO, United States, Cat: 69673), MD-TM mobile phase (Thermo-Scientific, Waltham, United States, Cat: 701332) were used for quantifying DA and its metabolites.

### Treatment of flies

Treatment of the flies with PQ was done as described by Phom et al. (2014). Whatman filter paper No.1 was used for disc-feeding experiments. Briefly, 10 mM PQ was prepared in 5% sucrose solution, and 275 μL of the solution was poured on filter paper. Twenty-five flies of the same age groups were placed in each vial. The climbing ability was noted every 24 h.

### Negative geotaxis assay

A negative geotaxis assay (climbing assay) was performed as described by Botella et al. (2008) and Phom et al. (2021). Briefly, an individual fly was placed into the plastic tube and given 2 min to acclimatize. The fly was then taped to the bottom of the tube, and the height it climbed in 12 s was recorded. A minimum of 12 flies were scored for each group, and the experiment was performed three times with each fly. The time point at which the fly showed significant mobility defect but not mortality was chosen to analyze the DAergic neuronal system.

### Immunostaining of the whole *Drosophila* brain

Quantification of DAergic neurodegeneration of the fly brain was done as described in Bayersdorfer et al. (2010) and Ayajuddin et al. (2022). Elaborately, the brains of male Oregon K flies were fixed in 4% paraformaldehyde (PFA) containing 0.5% TritonX (TX)-100, at room temperature for 2 h, and then washed five times after every 15 min (5 × 15 min) in phosphate-buffered saline (PBS) with 0.1% TX-100 (PBST), at room temperature (RT). Blocking was performed using PBS containing 0.5% TX-100 and 5% normal goat serum (NGS) for 120 min at room temperature (RT). Then, primary antibody (anti-TH) incubation was done for the brains with a dilution of 1:250 for 72 h at 4°C. The excess primary antibody was washed off from the brains for 5 × 15 min with PBST. Brains were then incubated with 1:250 dilution of secondary antibody (TRITC labeled) for 24 h at RT under dark conditions. After thorough washing for 5 × 15 min in PBST to remove the excess secondary antibodies, brains were mounted in VECTASHIELD® mounting medium and then topped with cover glass (Electron Microscopy Sciences, PA, United States), and image acquisition was done on the same day.

### Image acquisition

Prepared/stained brains were viewed under a fluorescence microscope (Axio Imager M2 with 100 W Mercury lamp, Carl Zeiss, Germany) at a 40 X lens (Supplementary Figure S1). The image was scanned using a monochromatic camera with a Rhodamine filter (Supplementary Figure S2). The image acquisition at 40X was done by using a red dot test for visibility of neuron(s) and assessing saturation using the control brain and reusing the same exposure time for all other brain samples (Supplementary Figure S2). Then, Z-stack programming with constant intervals was performed (Supplementary Figure S3). For image processing in 2D, on the method column, image subset and maximum intensity projection (MIP) with X–Y Plane was created (Supplementary Figure S4). Supplementary Figure S5 illustrates the merged fly brain image in 2D, which was used for presentation.

### Fluorescence intensity quantification

FI quantification is performed using ZEN 2012 SP2 software from Carl Zeiss, Germany. ZEN 2012 SP2, Carl Zeiss software is a single user and a license must be acquired to utilize the imaging system to interactively control image acquisition, image processing, and analysis. The FI quantification was done using 3D scan images. Briefly, PAL, PPL1, PPL2, PPM1/2, PPM3 (PAL, Protocerebral anterior lateral; PPL, Protocerebral posterior lateral; PPM, Protocerebral posterior medial) brain regions were selected (Supplementary Figure S6). The images were enlarged to see clear neurites, then from graphics appropriate tools draw spline contour were selected and a line was drawn around the neuron giving intensity mean and area (Supplementary Figure S7) and intensity sum was created by selecting more options (Supplementary Figure S8). From the measure tab on the left side of the panel; the list, view all, and create document options were selected (Supplementary Figure S9). The area and FI sum were recorded for each scan of a neuron in .xml format (Supplementary Figure S10). For quantification of FI of a single neuron, a total of 11 scans with an interval of 1.08 μm for each scan, meaning the cumulative of 11.08 μm width was considered (Supplementary Figure S11). The same process was followed for all the neurons located in different clusters. The intensity sum of all the neurons in a specific cluster gives the total FI of that particular region (cluster-wise). The total FI is the sum of the FI of all the neurons belonging to all the **DAergic neuronal** clusters. The fly brain with the same orientation was carefully chosen for FI quantification.

### Protein extraction from *Drosophila* brains

Fifty fly heads per group were homogenized in 160ul of RIPA buffer (50 mM Tris HCL, 1% Triton, 0.5% sodium deoxycholate, 150 mM NaCl, 0.1% SDS, 2 mM EDTA) with protease inhibitor cocktail (10% working concentration). Homogenates were then sonicated for 20s (with a pulse of 10s and amplitude at 30%) using a sonicator (Qsonica, OHIO industries, OH, United States). The samples were centrifuged at 13,000 rpm for 5 min at 4°C. The supernatant was re-centrifuged at 13,000 rpm for 5 min at 4°C.

### Protein quantification

Protein quantification was performed using the Bio-Rad RCDC assay reagent. BSA at a concentration of 1 mg/mL was used as the standard and 5 uL of the extracted protein lysates were used for quantification of the samples. Absorbance was read at 750 nm using the NanoDrop 2000C (Thermo Scientific, MA, United States). Lysates were stored at −80°C until further use.

### Western blotting

#### Bio-Rad proprietary stain-free western blotting

The casting of Bio-Rad stain-free gels: SDS-polyacrylamide gels were cast using the Bio-Rad TGX stain-free fast-cast acrylamide kit (10% and 1.5 mm thickness). In brief, the procedure is as follows: Resolving gel solution is prepared by mixing equal volumes of resolver A and resolver B solution (as described by the manufacturer). Added TEMED and freshly prepared 10% APS to the combined resolver, mixed well, and dispensed the solution into the glass plates. Filled the cassette to 1 cm below the bottom of the teeth of the comb. Then prepared stacking gel acrylamide solution by combining equal volumes of stacker A and stacker B solution (as described by the manufacturer). Added the TEMED and 10% APS to the stacker solution, mixed well, and pipetted the solution in the middle of the cassette, filling to the top of the plate (applied slowly and steadily to prevent mixing with resolving solution). Allowed the gel to polymerize for 30–45 min before starting the run.

### Preparing the fly brain protein lysate for SDS-PAGE

40 ug of each sample lysate was mixed with 20 uL of sample buffer (0.5 M Tris HCl pH6.8, 10%SDS, glycerol, 0.1% bromophenol blue, β-mercapto ethanol) in a total volume of 40 uL and denatured for 5 min at 95°C. A 10X stock of running buffer (Tris, Glycine, and SDS) was used for preparing a 1X running buffer. The gel was run at a current of 20mAmp at room temperature using a Bio-Rad powerpack basic power system. Before electro-blotting, the stain-free gels were scanned and activated (2.5 min) using the Bio-Rad fluorescent documentation system. PVDF membrane was used after activation by methanol before setting up the transfer sandwich. The transfer was carried out at a voltage of 90 V for the duration of 90 min using chilled 1X transfer buffer (Glycine, Tris, and methanol) with continuous stirring using a magnetic bead. To create a cold temperature condition the transfer tank was placed inside a bucket filled with ice.

### Membrane blocking and antibody treatment

Post-transfer the PVDF membrane was scanned using the Bio-Rad fluorescent documentation system and incubated in the blocking buffer of 5% BSA in 1X TBS-T (0.05%) for a duration of 90 min at room temperature with gentle rocking. Rabbit polyclonal anti-TH antibody was used in the dilution of 1:1,000 and the membrane was incubated at 4°C for 48 h. Post-primary antibody incubation, the membrane was washed 3X in 1X TBS-T (0.05%) for 15 min at room temperature. The secondary antibody Goat anti-rabbit HRP was used in a dilution of 1:5,000 and the membrane was incubated for 24 h at 4°C. The membrane was washed 5X in 1XTBS-T (0.05%) for 15 min and developed using ECL substrate. Scanning was performed with Bio-Rad fluorescent documentation system. TH protein amount was quantified using the whole protein normalization method (using Bio-Rad proprietary stain-free gels) that requires no loading control. Data analysis was performed using Bio-Rad ImageLab 5.2.1 version software.

(Details of WPN (whole protein normalization method) and calculation of TH protein amount in a brain sample are presented in Supplementary material 2).

### Quantification of DA and metabolites (DOPAC and HVA) using high-performance liquid chromatography with an electrochemical detector

Brain-specific DA and its metabolites were quantified using high-performance liquid chromatography with an electrochemical detector (HPLC-ECD; HPLC-Thermo Scientific, Dionex Ultimate 3000) following the protocol described by Ayajuddin et al. (2021). The control group and PQ-exposed group of flies were immediately frozen following 24 h of exposure. To avoid thawing of tissue and degradation of biomolecules, frozen flies were placed on an ice tray containing a chilled metal surface and 15 fly heads were decapitated quickly with a sharp scalpel. Head tissue homogenate was prepared in 300 μL of chilled PBS. Sonication of the homogenate was performed at 30% amplitude for 20 s with 5-s intervals. Then homogenate was centrifuged at 6,000 rpm, 4°C for 10 min. After centrifugation, 50 μL of the supernatant was set aside for protein quantification. The remaining was combined with 5% TCA (prepared in HPLC grade or enzyme-free water) in a 1:1 ratio and kept in ice. Standard DA, DOPAC, HIAA, and HVA were prepared in PBS, each having a concentration of 200 ng/mL. The standard solution was mixed with 5% TCA in a 1:1 ratio and kept on ice to prevent catecholamine degradation. For quantification, 50 μL of the tissue sample and 20 μL of the composite standard were loaded into the HPLC. MCM 15 cm × 4.6 mm, 5 μ C-18 packed columns (Thermo-Scientific, Waltham, United States, Cat: 70-0340) was used as the stationary phase for elution of the catecholamines, and MD-TM served as the mobile phase. To detect the catecholamines, the reduction and oxidation potentials within the two cells of primary ECD were kept at −175 and +225 mV, respectively. The secondary ECD module acting as a third cell also known as Omnicell, was set to +500 mV to reduce background noise. Data was gathered at a rate of 5 Hz. Chromatogram analysis was done using Chromeleon® 7 from Thermo-Scientific (Waltham, United States). Comparisons were made between sample and standard chromatograms for a catecholamine’s retention time. To precisely pinpoint the peaks corresponding to DA, DOPAC, and HVA in the sample, 10 μL of the composite standard was added to a sample and run through the HPLC once again. The spiked peaks according to the detection sequence in the standard solution were recognized as the catecholamines of interest in the sample.

Quantification and normalization of catecholamines were described by Ayajuddin et al. (2021).

In brief, (1) The concentration of a catecholamine is: C_Std_ (ng/mL), (2) the area of a catecholamine in the composite standard chromatogram is: A_Std,_ and the injection volume of the composite standard solution is: I_Std_ (μL), (3) the area of the catecholamine in the tissue extract chromatogram is: A_Samp_ and the injection volume of the tissue extract is I_Samp_ (μL), (4) total number of fly heads for protein extraction: N, (5) the total protein concentration of the tissue extract is: P_Samp_ (μg/μL).

Calculation steps (Supplementary material 3):

1. The standard catecholamine concentration in I_Std_ (μL) injection volume: (C_Std_ X I_Std_)/1,000 = V1 (ng).
2. The catecholamine concentration in tissue extract: (A_Samp_ X V1)/A_Std_ = V2 (ng).
3. Total protein in I_Samp_ (μL) injection volume of tissue extract: (P_Samp_ X I_Samp_) = V3 (μg).
4. The catecholamine concentration per 1 μg in the injected tissue extract: V2/V3 = V4 (ng/1 μg).
5. The catecholamine concentration per fly head = V5/N = V6 (ng).
6. Injected tissue extract and the standard solution was mixed with 5% TCA in a 1:1 ratio. Therefore, the actual catecholamine concentration per fly head (V6/2) = V7 (ng) or (V7 X1000) = V8 (pg).

(Detailed calculation of the concentration of catecholamines in a brain sample with an example is presented in Supplementary material 3).

### Data analysis

GraphPad Prism 5.0 software was used to perform statistical analysis and preparation of the graphs, which were expressed as mean ± standard error of the mean (SEM). For the two-group data, statistical significance was determined by a two-tailed unpaired t-test. A one-way analysis of variance (ANOVA) followed by the Newman–Keuls Multiple Comparison Test was carried out for the data with more than two groups. *p*-value < 0.05 was considered significant.

## Results

### PQ induces Parkinsonian symptoms as indicated by negative geo-taxis assay

**Figure 1:**
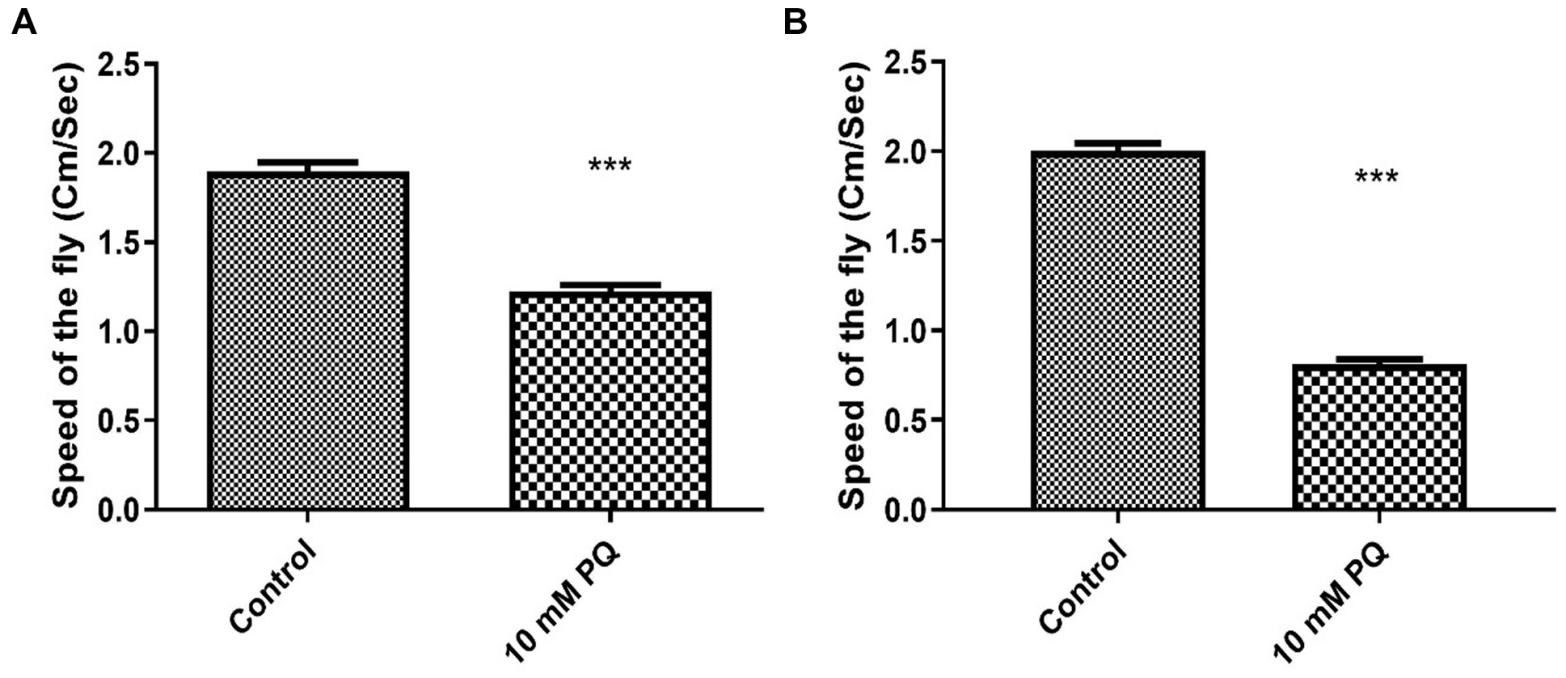
Assessing the climbing ability of *Drosophila* by negative-geotaxis assay after exposure to PQ: the climbing speed of flies within 12 s was assayed. Ingestion of 10 mM PQ caused severe mobility defects after 24 **(A)** and 48 h **(B)** of exposure. Mobility defects enhanced with exposure duration, but no mortality was observed for the exposure duration of 24 and 48 h. Hence, an exposure window of 24 h was selected for further experiments. Unpaired *t*-test reveals a significant reduction in mobility of PQ exposed fly compared to control. ***p < 0.001.

A PQ-induced fly model of sporadic PD was developed in our laboratory (Phom et al., 2014) and the same is employed in the present study. Flies were fed with 10 mM PQ prepared in 5% sucrose to induce PD, whereas the control flies remained in 5% sucrose. The negative geotaxis assay was used to measure mobility impairments. Results revealed that 90% of the flies could reach the top of the column in 12 s under normal conditions, but PQ-treated flies were unable to do so. PQ-intoxicated *Drosophila* exhibited resting tremors and bradykinesia, which are the distinctive clinical symptoms of PD in human patients. The flies failed to hold their grip and slipped to the bottom as they tried to climb on the wall. Further, the climbing speed of the flies declined by 33% after 24 h and by 60% after 48 h of exposure to the neurotoxicant (Figures 1A,B). No mortality was observed after either 24 or 48 h of exposure window, yet flies showed significant mobility defects. Therefore, an exposure window of 24 h was chosen to characterize the DAergic neurodegeneration.

### Fly model of PD shows no loss of DA neurons but depletion in TH synthesis: insights from whole brain immunostaining and western blotting

**Figure 2:**
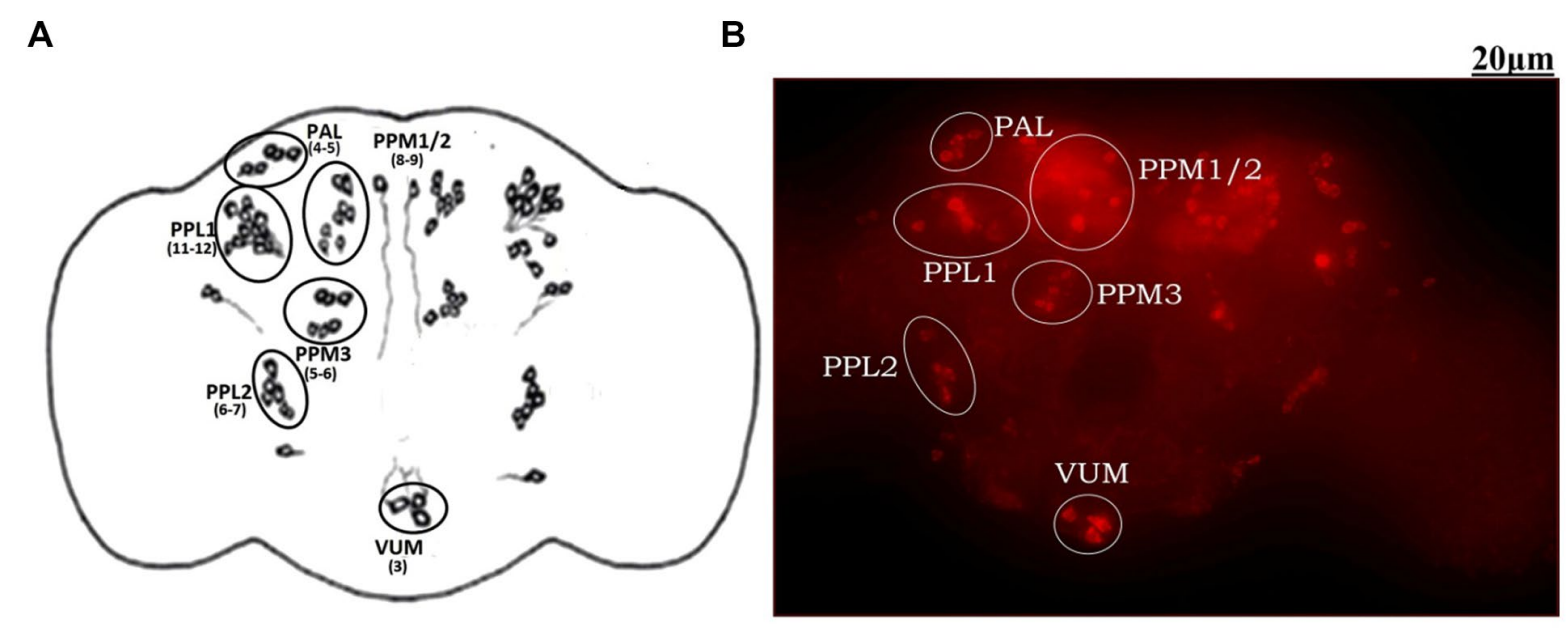
Demonstration of DAergic neurons in the whole fly brain: cartoon of *Drosophila melanogaster* brain illustrating the position of quantifiable DAergic neurons **(A)** and image of the whole-brain mount of 5 days old male *Drosophila* captured using *ZEN software* of Carl Zeiss Fluorescence Microscope using fluorescently labeled secondary antibody targeted against the primary anti-TH antibody **(B)**. In the fly brain, ~140 DAergic neurons (including ~100 neurons of the PAM cluster which cannot be quantified due to the high neuronal density) are arranged in different clusters in each hemisphere. The Scale bar of the brain image in the panel is 20 μm (PAL, Proto-cerebral Anterior Lateral; PAM, Proto-cerebral Anterior Medial; PPL, Proto-cerebral Posterior Lateral; PPM, Proto-cerebral Posterior Medial; VUM, Ventral Unpaired Medial).

**Figure 3:**
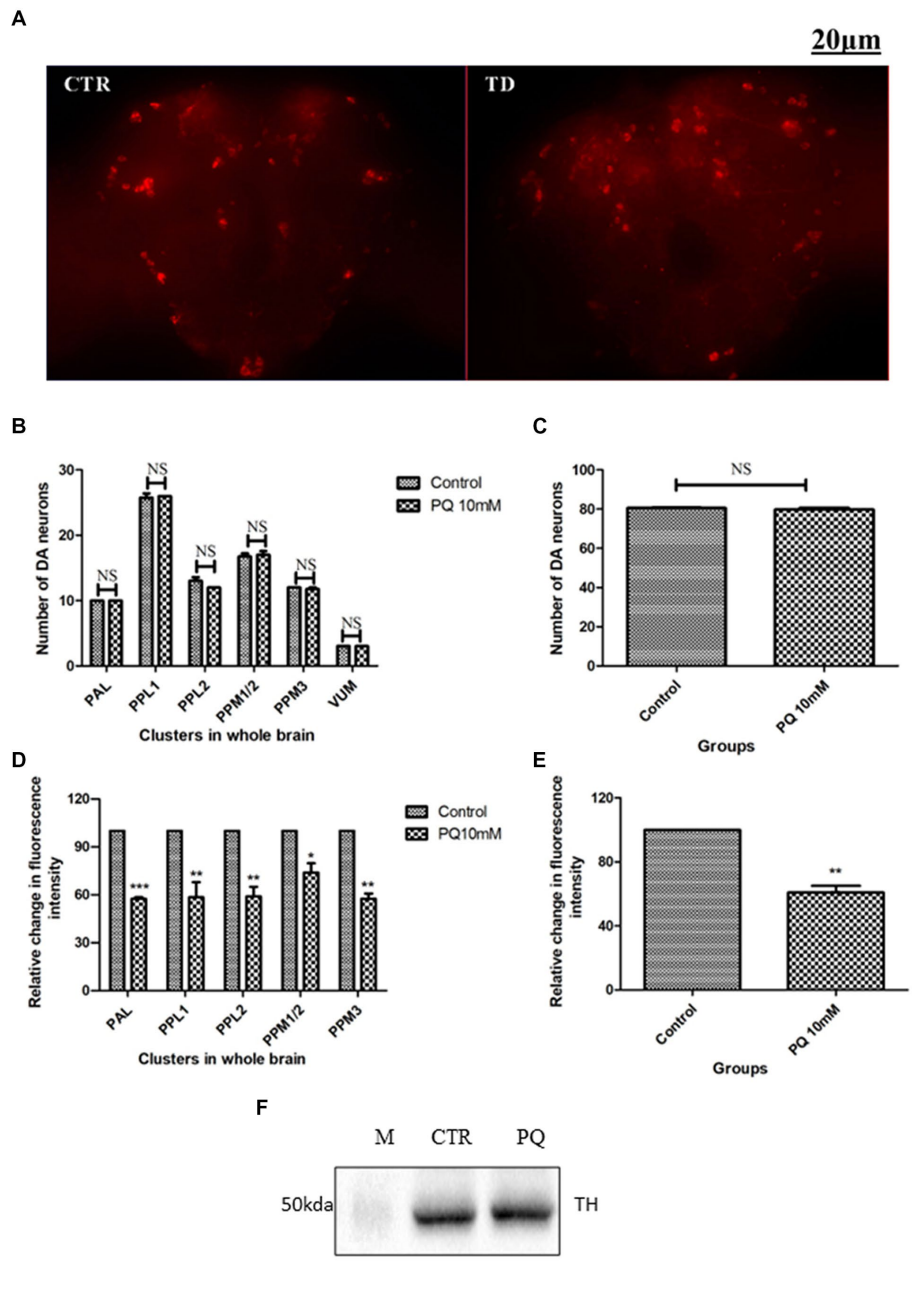
Characterization of DAergic neurodegeneration in the whole fly brain through anti-TH antibody immunostaining and quantification of brain TH protein using western blotting: the image depicts the whole brain mount of the adult *Drosophila* under control and PQ-treated conditions **(A)** (CTR, Control; TD, PQ-treated). Quantification of DAergic neurons reveals that the neuronal number remains unaffected **(B,C)**, whereas the fluorescence intensity (of fluorescently labeled secondary antibodies that target primary antibody anti-TH) is significantly decreased in all the clusters **(D)**, and *in toto* **(E)** between the control and treated group. The scale bar of the brain images in the panel is 20 μm. (CTR, Control; TD, Treated with 10 mM PQ; Represented images are “merged” Z-stacking images; however, the quantification of DA neuronal number and fluorescence intensity is performed in 3D Z-stack images; PAL, Protocerebral anterior lateral; PPL, Protocerebral posterior lateral; PPM, Protocerebral posterior medial). **(F)** Stain Free Western Blot analysis shows a reduction of brain TH protein (15%) upon PQ treatment in the fly model of PD [Bio-Rad Stain-Free Western Blotting using total protein normalization method (TPN) (M-protein ladder; CTR-control; PQ- paraquat treated)]. Statistical analysis was performed using a *t*-test (compared to control). *p < 0.05, **p < 0.01, ***p < 0.001; NS, not-significant.

The adult *Drosophila* brain comprises six countable DAergic neuronal clusters in each hemisphere of the brain (Figures 2A,B). To probe into DAergic neuronal dysfunction in PQ-administered flies, the brains were immuno-stained for TH (Figure 3A). Quantification of DAergic neuronal number reveals no significant difference in all the five clusters analyzed, and *in toto* in PD-induced brains as compared to the control (Figures 3B,C). However, there are slight changes in the neuronal number even among the control groups, which can be accredited to natural variation. This result is consistent with the earlier comprehensive study performed by Navarro et al. (2014). To comprehend whether there is a change in the level of TH synthesis, the FI of the DAergic neurons (fluorescently labeled secondary antibody (ab) targets the primary anti-TH ab, and hence FI is correlated to the level of TH protein synthesis) was quantified. In PQ-administered flies, the FI of DAergic neurons in the PAL, PPL1, PPL2, and PPM3 clusters acutely decreased except for PPM1/2 (Figure 3D). The FI of DAergic neurons (five quantifiable DA neuronal clusters) of the whole brain mount exhibited a significant reduction (30%–35%) as compared to the control group (Figure 3E). Further, by employing the Bio-Rad Stain-Free Western Blotting (BR-SFWB), fly brain protein lysate was probed to quantify the level of TH protein (BR-SFWB enables total protein normalization method and requires no loading control). Results illustrate a reduction in brain TH protein upon PQ treatment (15% depletion; Figure 3F). Immunostaining together with western blotting, results elucidate that in the fly PD model, though there is no loss in the number of DA neurons, the TH protein synthesis is diminished suggesting the “neuronal dysfunction.”

### PQ exposure diminishes brain DA and enhances its oxidative turnover rate

**Figure 4:**
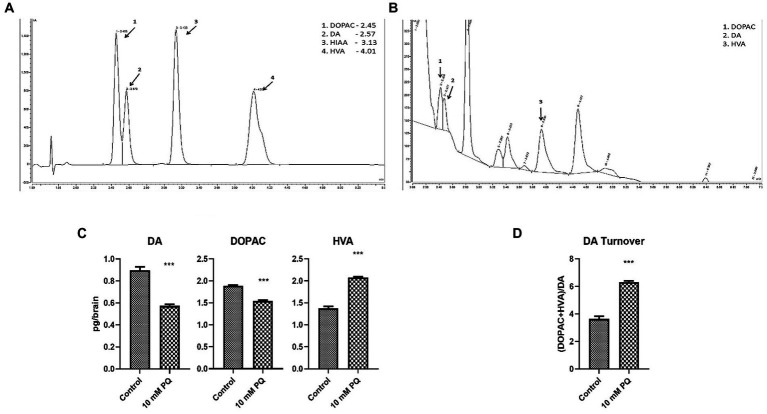
Quantification of DA and its metabolites (DOPAC, HVA) in fly brain tissue extract using HPLC-ECD: the retention time of standard DA, DOPAC, and HVA is shown in the chromatogram **(A)** and chromatogram for the fly head tissue extract shows the detected catecholamines **(B)**. Quantification of the brain-specific catecholamines revealed that PQ exposure for 24 h depleted brain DA and DOPAC levels, whereas the HVA level is significantly enhanced **(C)**. The result also revealed that in the induced PD condition there is a higher oxidative turnover of DA to its metabolites (DOPAC and HVA) **(D)**. Statistical analysis was performed using an unpaired *t*-test (compared to the control), *** *p* < 0.001.

TH is the rate-limiting enzyme in the synthesis of DA. Therefore, to further understand the implication of diminished TH synthesis in the brain, the DA level was also quantified (Figures 4A,B). The result demonstrated that PQ exposure diminishes brain DA levels (Figure 4C), which can be attributed to diminished TH synthesis under PD conditions. Further, oxidative turnover of DA is also linked with the onset of Parkinsonian symptoms. Therefore, downstream metabolites of DA, i.e., DOPAC and HVA levels were also assayed. The result revealed that the neurotoxicant exposure promoted DOPAC depletion which was not as much as DA depletion (Figure 4C), whereas the HVA level was enhanced in the induced PD condition (Figure 4C). This observation signified enhanced oxidative turnover of DA under the induced PD condition (Figure 4D). Insight suggests that the onset of Parkinsonian symptoms underlies the depletion of TH synthesis in DAergic neurons contributing to diminished DA levels in the fly brain. Further, such induced PD conditions also promote oxidative turnover of DA.

## Discussion

Two methods have been usually used in *Drosophila* models to envisage the DAergic neuronal system: (1) anti-TH immunostaining or (2) the targeted over-expression of a GFP reporter gene under the control of either TH-GAL4 or Ddc-GAL4 transgenes. The pattern of TH-GAL4 expression and anti-TH immunoreactivity in the adult protocerebrum was originally described in Nässel and Elekes (1992) and Friggi-Grelin et al. (2003) and further characterized by Mao and Davis (2009) and White et al. (2010).

The negative geotaxis assay clearly shows a significant decrease in climbing ability showing Parkinsonian symptoms which might be induced by DAergic neurodegeneration (Figure 1). But, the numbers of DAergic neurons remain unchanged as we count all the clusters which is consistent with other studies (Meulener et al., 2005; Navarro et al., 2014).

Loss of DAergic neurons *per se* in fly PD models has been an issue of debate and it was re-evaluated in both the sporadic and genetic fly models of PD and resolved that there is no loss of DAergic neurons; however, there is a diminished level of TH synthesis (Navarro et al., 2014). In the present method, we have used anti-TH immunostaining. We found that the number of DAergic neurons in the whole fly brain under PQ-induced PD condition remains unchanged. Nevertheless, the FI of the secondary antibody which is targeted against the primary anti-TH antibody is diminished suggesting decreased TH synthesis. Decreased TH protein synthesis is further confirmed through western blotting.

Quantification of TH protein using whole brain immunostaining reveals that it is about 30–35% depletion in the PQ-treated brain and the western blotting result illustrates that it is a 15% reduction. The observed variations in the level of TH depletion can be attributed to the following reasons:

1. It can be due to the variation/limitation in the sensitivity levels of the two methods.
2. It is important to note that the quantification of FI was limited to only five quantifiable clusters in the whole fly brain (quantifiable five clusters together constitute ~40 neurons only out of a total of ~140 DAergic neurons in one hemisphere of the fly brain! ~ 100 neurons of the PAM cluster cannot be quantified due to the high neuronal density). Whereas western blotting is done in whole brain protein lysate (that includes all the DA neuronal clusters).
3. It is worth mentioning that it has been demonstrated in fly models of PD that the extent DAergic neurodegeneration varies among different DA neuronal clusters and degeneration is also can be cluster-specific (Coulom and Birman, 2004; Chaudhuri et al., 2007; Wang et al., 2007; Maitra et al., 2021; Chaouhan et al., 2022).

This approach allowed us to accurately assess the neurodegeneration of DAergic neurons in the fly PD model. While using this methodology, it was comprehended that the orientation of the brains mounted for fluorescence microscopy could impact the quantification of FI of DAergic neurons. Therefore, for an optimal imagining of the DAergic neuronal clusters, neurons that confine to the posterior protocerebrum were taken into consideration, and slightly damaged or torn brains were not examined. In this study, all brains were analyzed in the same orientation.

Auluck et al. (2002) studied both the paraffin sections and the whole brain mount. They observed a change in the number of DAergic neurons in the PPM1/2 cluster in the paraffin section which is not observed in the whole brain mount showing the unreliability of the previous method. Using fluorescence microscopy, we quantified both the DAergic neuronal number and FI. Results reveal that FI was significantly down-regulated under PQ-induced conditions as compared with controls (Figure 3E). The result was consistent with the observed FI of GFP reporter genes (Navarro et al., 2014). Grasping the idea from their study, we have assessed the neuroprotective efficacy of curcumin in the *Drosophila* model of PD (Ayajuddin et al., 2022), which indicates the reliability of the current method of quantification.

TH is the rate-limiting enzyme for DA synthesis. Therefore, to further validate the current methodology we also quantified the levels of brain DA and its metabolites (DOPAC and HVA). The result demonstrated that the induced PD condition depleted brain DA levels (Figure 4C). Diminished brain DA level is a prime characteristic of PD in human and PQ-induced animal models of PD (Goldstein et al., 2011; Phom et al., 2014). It has been demonstrated that oxidative turnover of DA to DOPAC and HVA contributes to the onset of PD, owing to the neurotoxic nature of the process (Zhang et al., 2019; Cao et al., 2021). Observation in the current study demonstrated that DA turnover is increased in the induced PD condition (Figure 4D) which further explains the enhanced oxidative stress and neurotoxicity under the induced PD condition. Insights from the catecholamine quantification with the HPLC-ECD method corroborate neurophysiological changes associated with PD and further validate the reliability of the FI quantification method.

Demonstration of mobility defects, reduced FI of DA neuronal clusters, reduction in TH protein synthesis, depleted brain DA, and enhanced DA turnover phenotypes illustrates the robustness of the present method of quantification of DAergic neurodegeneration through quantification of FI using fluorescence microscopy.

Fluorescence microscopy, on the other hand, is much simpler to handle, easier to operate, and also gives precise results whereas confocal microscopy is cumbersome and costlier. As we do not have access to expensive confocal microscopy, we worked on and developed this method which can be of use to neurobiologists working in fund-constrained academic institutions of developing countries.

Through this assay, it is possible to characterize the incipient DAergic neurodegeneration that will be of great support in following the progression of the disease which is a critical necessity to screen novel neuroprotective molecules, in order to develop smart and successful therapeutic strategies.

## Data availability statement

The original contributions presented in the study are included in the article/Supplementary material, further inquiries can be directed to the corresponding author.

## Author contributions

MA, RC, and ZK performed experiments relating to whole-brain immunostaining. MA, AD, and LP performed experiments relating to the quantification of DA and its metabolites using HPLC-ECD. PM performed experiments relating to the quantification of brain TH protein through western blotting. AD conducted PQ treatments and performed fly mobility experiments. MA, AD, PM, and RC involved in acquisition, analysis of the data, and manuscript drafting. SY contributed to conception and design of the study, obtained funding, involved in interpretation of the data, manuscript revision, and supervision. All authors contributed to the article and approved the submitted version.

## Funding

This research was supported by the Department of Biotechnology (DBT), India (R&D grant nos. BT/405/NE/U-Excel/2013 and BT/PR16868/NER/95/328/2015) and the Science and Engineering Research Board (SERB) of the Department of Science and Technology (DST) India (R&D grant no. EMR/2016/002375, 27-3-2017). LP, MA, PM, AD, and RC received the DBT-JRF (Junior Research Fellowship); LP, PM, AD, and RC received DBT-SRF (senior research fellowship); ZK received MANF (Maulana Azad National Fellowship) of the Ministry of Minority Affairs, India; and MA and RC received ICMR (Indian Council of Medical Research)-SRF.

## Conflict of interest

The authors declare that the research was conducted in the absence of any commercial or financial relationships that could be construed as a potential conflict of interest.

## Publisher’s note

All claims expressed in this article are solely those of the authors and do not necessarily represent those of their affiliated organizations, or those of the publisher, the editors and the reviewers. Any product that may be evaluated in this article, or claim that may be made by its manufacturer, is not guaranteed or endorsed by the publisher.
