# Supplementary material for: Fluorescence microscopy-based sensitive method to quantify dopaminergic neurodegeneration in a Drosophila model of Parkinson’s disease
Source: Front Neurosci. 2023 Jun 26;17:1158858. doi: 10.3389/fnins.2023.1158858 (PMC10332464; doi:10.3389/fnins.2023.1158858)
Supplement: Supplementary file 1 [file Data_Sheet_1.PDF]

Supplementary 2

Fluorescence Microscopy-Based Sensitive Method to Quantify Dopaminergic Neurodegeneration in a *Drosophila* Model of Parkinson's Disease

Mohamad Ayajuddin[1]†, Rahul Chaurasia[1]†, Abhik Das[1]†, Priyanka Modi[1]†, Limamanen Phom[1,2], Zevelou Koza[1,3], and Sarat Chandra Yenisetti[1]*

Bio-Rad Proprietary Stain-Free Gel Western Blotting: (whole protein normalization method)

Data analysis was done using the ImageLab 5.2.1 version software.

Normalization with stain-free blot and expression analysis of Tyrosine hydroxylase

1. Open the images of stain-free blot and chemi blot (image for representative purpose only)

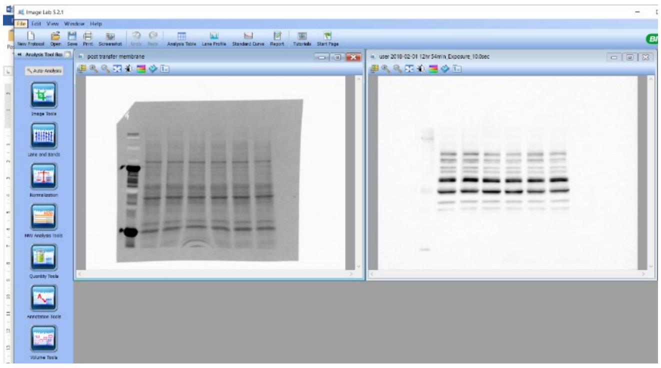

2. Select a multichannel image option

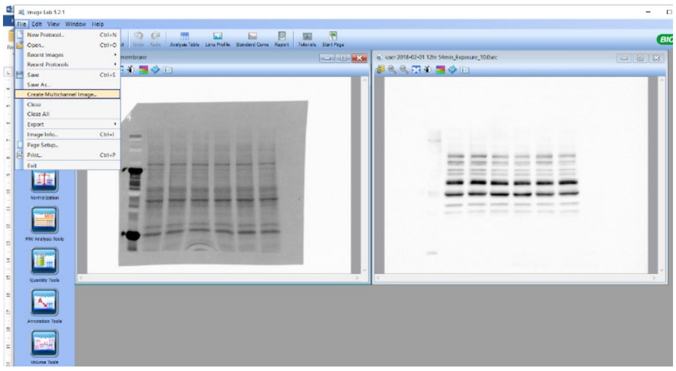

3. **Quantification of brain TH expression in the present experiment:**

a. Remove the RBG and on the stain-free blot select lanes by manually selecting the number of lanes

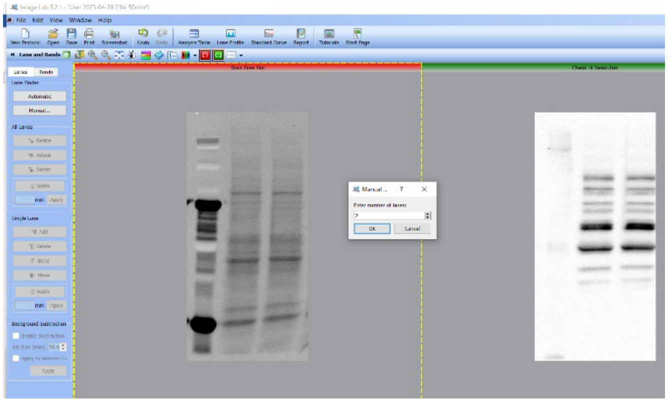

b. Adjust and resize the lanes. Copy all the lanes from the stain free and paste it onto the chemi blot. Adjust and resize the lanes on the chemi blot

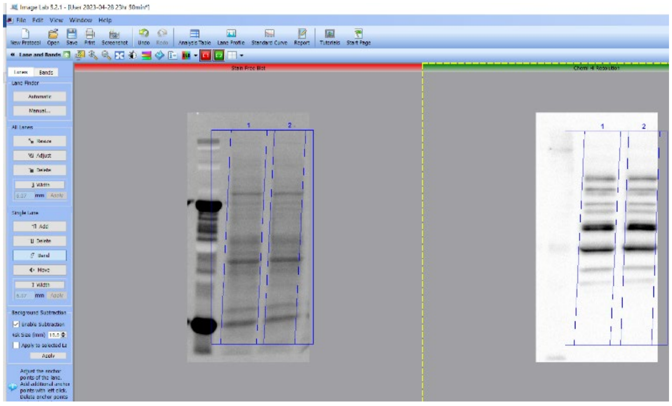

c. Select bands and click add bands on the chemi blot. Add one band per lane. Adjust the boundary and anchors of the bands on each lane so that it encompasses the whole length of the respective lanes.

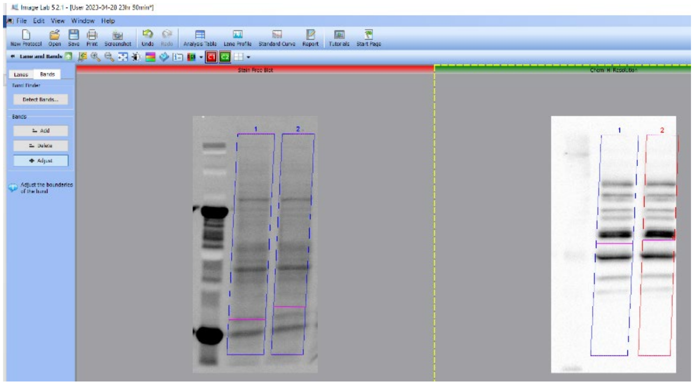

d. Select normalization on the tool panel and select stain-free blot as the normalization channel

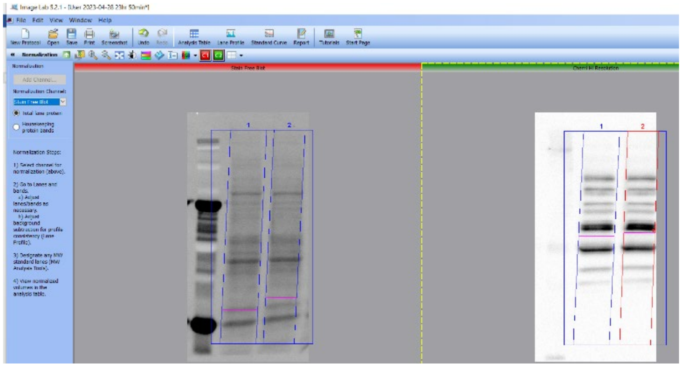

e. Click on the analysis table to get the normalization factor for each lane on the chemi blot

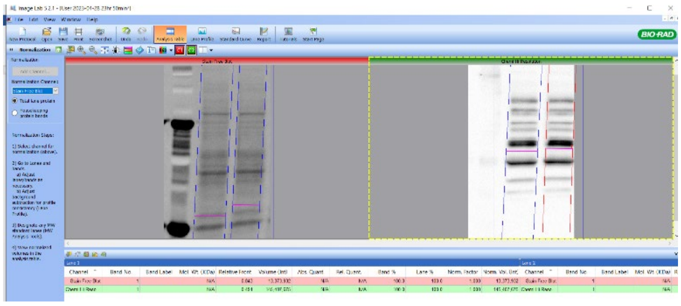

f. From the file, click export and select lane and bands table to excel

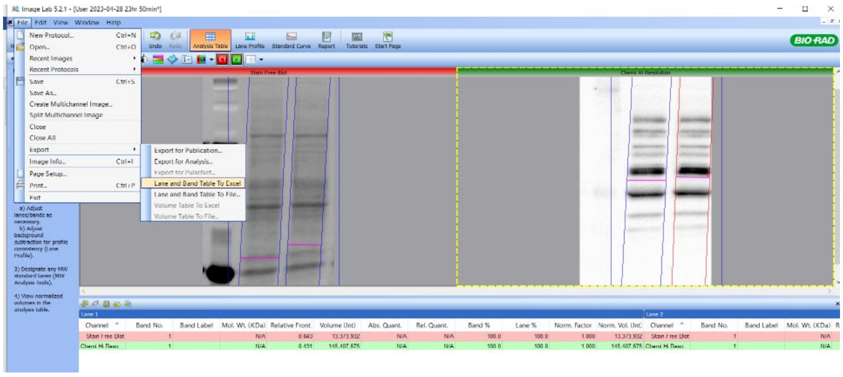

g. Excel sheet for normalization factor values for the chemi blot

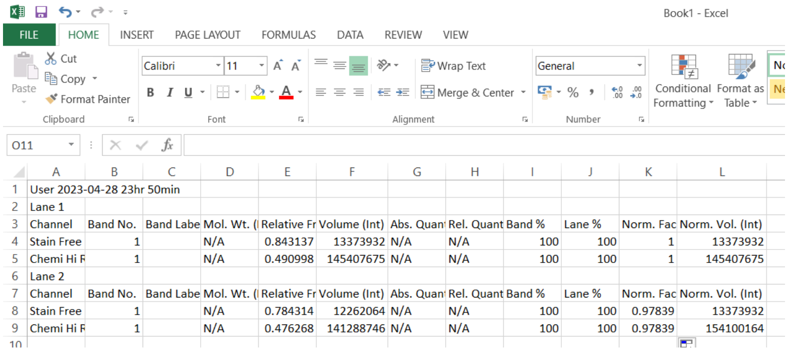

	A	B	C	D	E	F	G	H	I	J	K	L
1	User 2023-04-28 23hr 50min											
2	Lane 1											
3	Channel	Band No.	Band Labe	Mol. Wt. (	Relative Fr	Volume (Int)	Abs. Quan	Rel. Quant	Band %	Lane %	Norm. Fac	Norm. Vol. (Int)
4	Stain Free	1		N/A	0.843137	13373932	N/A	N/A	100	100	1	13373932
5	Chemi Hi F	1		N/A	0.490998	145407675	N/A	N/A	100	100	1	145407675
6	Lane 2											
7	Channel	Band No.	Band Labe	Mol. Wt. (	Relative Fr	Volume (Int)	Abs. Quan	Rel. Quant	Band %	Lane %	Norm. Fac	Norm. Vol. (Int)
8	Stain Free	1		N/A	0.784314	12262064	N/A	N/A	100	100	0.97839	13373932
9	Chemi Hi F	1		N/A	0.476268	141288746	N/A	N/A	100	100	0.97839	154100164
10												

h. In the same multichannel, click on volume tools and select the rectangle and place it on the band of interest on the chemi blot. Copy and paste the same rectangle on the bands of interest in the other lanes. By default, they are labeled as unknown. Select the option lane from the volume tool and create a single lane and label it as background. Select local as the background subtraction method.

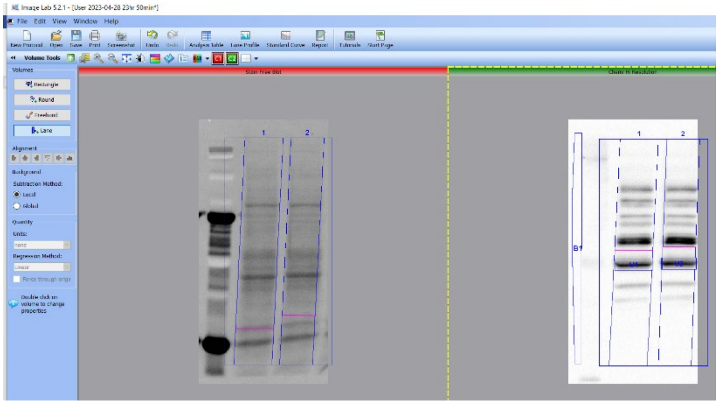

i.. Go to file. Select export and click on volume table to Excel.

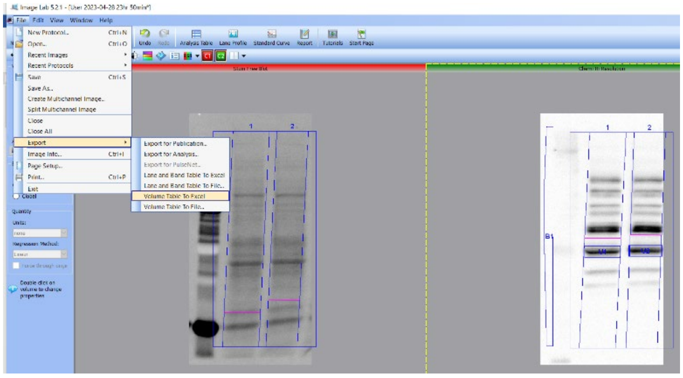

j. Excel file showing adjusted volume for all the lanes as well as the background.

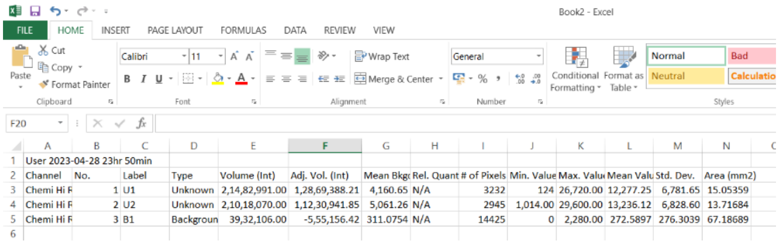

	A	B	C	D	E	F	G	H	I	J	K	L	M	N
1	User 2023-04-28 23hr 50min													
2	Channel	No.	Label	Type	Volume (Int)	Adj. Vol. (Int)	Mean Bkgr	Rel. Quant	# of Pixels	Min. Value	Max. Valu	Mean Valu	Std. Dev.	Area (mm2)
3	Chemi Hi R	1	U1	Unknown	2,14,82,991.00	1,28,69,388.21	4,160.65	N/A	3232	124	26,720.00	12,277.25	6,781.65	15.05359
4	Chemi Hi R	2	U2	Unknown	2,10,18,070.00	1,12,30,941.85	5,061.26	N/A	2945	1,014.00	29,600.00	13,236.12	6,828.60	13.71684
5	Chemi Hi R	3	B1	Backgroun	39,32,106.00	-5,55,156.42	311.0754	N/A	14425	0	2,280.00	272.5897	276.3039	67.18689
6														

k. In a new Excel sheet, add the adjusted volumes and calculate the expression of the band for each of the lanes as per the formula: **Normalized expression= Expression X NF.** Multiply the expression with the respective NF to give the normalized expression values for each lane (the first lane is the control lane).

Calculation of TH protein expression in Control and PQ-treated brains

STEPS	CONTROL	TREATED
Calculation of **Normalization Factor**: from the normalization tool of Bio-Rad ImageLab software.	1	0.97839
Calculation of **adjusted intensity**: band intensity as obtained from the volume analysis tool of the ImageLab software	1,28,69,388.21	1,12,30,941.85
**Expression** is calculated by dividing the adjusted intensity by the control adjusted intensity into all the groups (in the present study only two ie. Control and PQ-treated)	1,28,69,388.21/1,28,69,388.21 = 1	1,12,30,941.85/1,28,69,388.21 = 0.872686538
Calculation of **normalized expression**: multiply expression with the normalization factor	1X1=1	0.87268X0.97839= 0.85382

The result shows that upon PQ treatment depletion in brain TH protein is fifteen percent (15%) compared to control.
