# Supplementary material for: Fluorescence microscopy-based sensitive method to quantify dopaminergic neurodegeneration in a Drosophila model of Parkinson’s disease
Source: Front Neurosci. 2023 Jun 26;17:1158858. doi: 10.3389/fnins.2023.1158858 (PMC10332464; doi:10.3389/fnins.2023.1158858)
Supplement: Supplementary file 2 [file Data_Sheet_2.PDF]

Supplementary 3

Fluorescence Microscopy-Based Sensitive Method to Quantify Dopaminergic Neurodegeneration in a *Drosophila* Model of Parkinson's Disease

Mohamad Ayajuddin[1]†, Rahul Chaurasia[1]†, Abhik Das[1]†, Priyanka Modi[1]†, Limamanen Phom[1,2], Zevelou Koza[1,3], and Sarat Chandra Yenisetti[1]*

Calculation of concentration of catecholamines in a sample with Example:

(15 fly heads/group were used in the assay)

i. The concentration of the standard catecholamines: DA (DA_{Std}), DOPAC ($DOPAC_{Std}$), and HVA (HVA_{Std}) used in the HPLC assay was 200 ng/ml each.

ii. Injection volume of all standard catecholamines to the HPLC column was I_{Std} = 20 µl.

iii. Areas of the peak of the catecholamines (DA, DOPAC, and HVA) in the standard chromatogram were

A_{DA_Std} = 74.82, A_{DOPAC_Std} = 90.21 and A_{HVA_Std} = 112.13

iv. Injection volume of tissue extract to the column was I_{Samp} = 50 µl.

v. Areas of the peak of catecholamines (DA, DOPAC, and HVA) in the "Control" brain tissue sample chromatogram were A_{DA_Samp} = 3.02, A_{DOPAC_Samp} = 6.97, and A_{HVA_Samp} = 6.23.

vi. The brain tissue extract from the "Control" group that was used for the HPLC assay, was quantified beforehand for total protein which was TP_{Samp} = 0.114 µg/ µl.

vii. The following steps were followed for calculating the actual amount of the catecholamines in tissue extract (**Table 1**).

Calculation:

Calculation Steps	Metabolites		
	DA	DOPAC	HVA
Step I: Concentration of standard catecholamines in 20 µl of injection volume	DA_{Std} X I_{Std}/1000<br><br>i.e. (200 X 20)/1000 = 4 ng	$DOPAC_{Std}$ X I_{Std}/1000	HVA_{Std} X I_{Std}/1000<br><br>i.e. (200 X 20)/1000 = 4 ng

		i.e. (200 X 20)/1000 = 4 ng	
Step II: Concentration of catecholamines in brain tissue extract	$(A_{DA\_Samp}$ X 4)/ A_{DA_Std}<br><br>i.e. (3.02 X 4)/74.82 = 0.1615 ng	$(A_{DOPAC\_Samp}$ X 4)/ A_{DOPAC_Std}<br><br>i.e. (6.97 X 4)/90.21 = 0.3090 ng	$(A_{HVA\_Samp}$ X 4)/ A_{HVA_Std}<br><br>i.e. (6.23 X 4)/112.13 = 0.2222 ng
Step III: Determining the total protein in 50 µl that was injected into the column	$(TP_{Samp}$ X $I_{Samp})$<br><br>i.e. (50 X 0.114) = 5.7 µg	$(TP_{Samp}$ X $I_{Samp})$<br><br>i.e. (50 X 0.114) = 5.7 µg	$(TP_{Samp}$ X $I_{Samp})$<br><br>i.e. (50 X 0.114) = 5.7 µg
Step IV: Determining the catecholamine in 1 µg of total protein	0.1615/5.7 = 0.02833 ng	0.3090/5.7 =0.05421 ng	0.2222/5.7 = 0.03898 ng
Step V: Determining the actual amount of catecholamine as injected brain tissue extract and the standard solution had TCA in a 1:1 ratio	0.02833/2 = 0.014165 ng in 1 µg of total protein	0.05421/2 = 0.027105 ng in 1 µg of total protein	0.03898/2 = 0.01949 ng in 1 µg of total protein
Step VI: Determining the actual amount of catecholamine in each fly brain	(0.014165 X 1000)/15 = 0.94 pg	(0.027105 X 1000)/15 = 1.8 pg	(0.01949 X 1000)/15 = 1.29 pg

Table 1: Methodology for quantification of catecholamines in *Drosophila* brain tissue
