# Supplementary material for: Fluorescence microscopy-based sensitive method to quantify dopaminergic neurodegeneration in a Drosophila model of Parkinson’s disease
Source: Front Neurosci. 2023 Jun 26;17:1158858. doi: 10.3389/fnins.2023.1158858 (PMC10332464; doi:10.3389/fnins.2023.1158858)
Supplement: Supplementary file 3 [file Presentation_1.PPTX]

## Slide 1
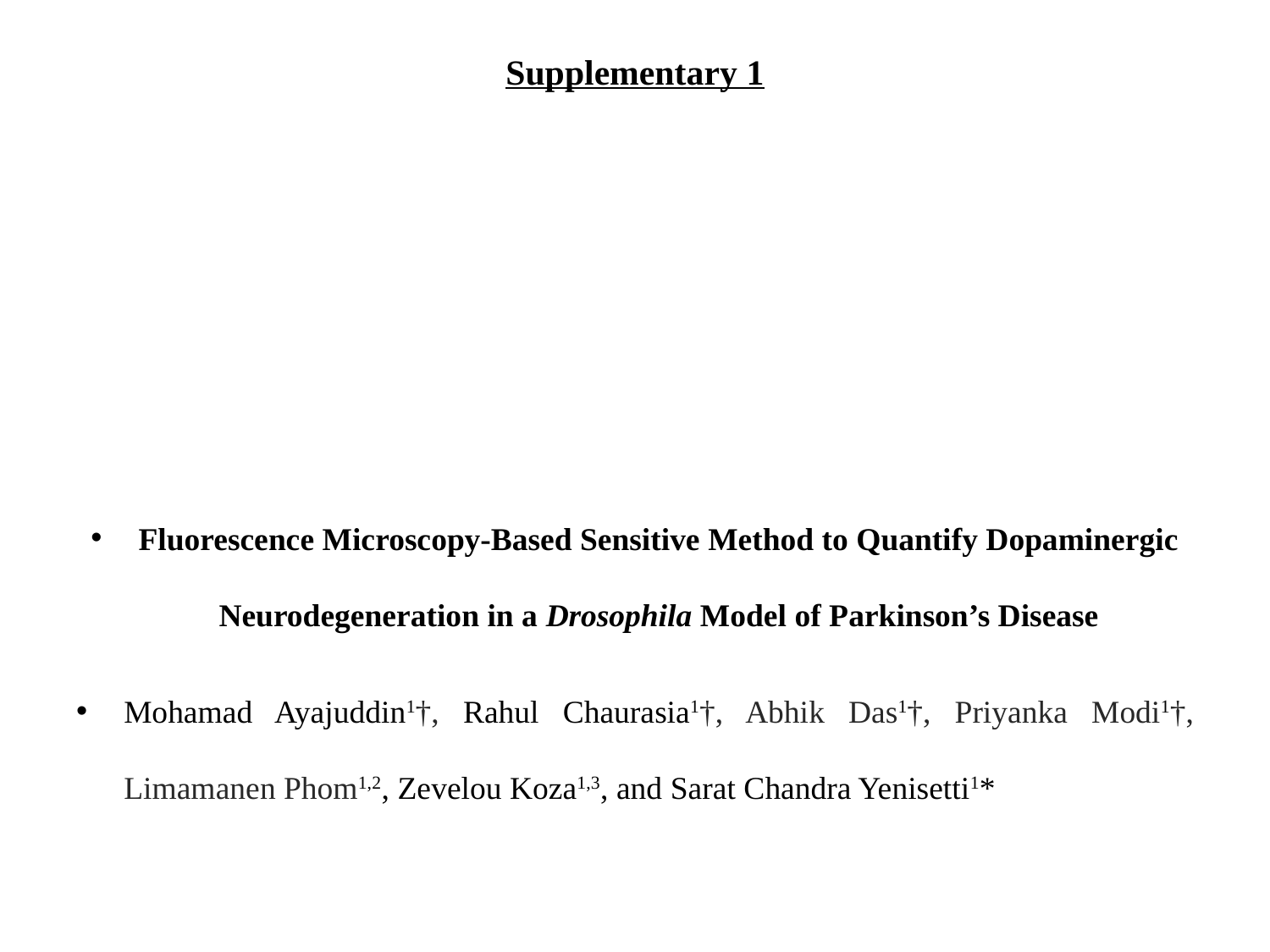

# Supplementary 1
Fluorescence Microscopy-Based Sensitive Method to Quantify Dopaminergic Neurodegeneration in a Drosophila Model of Parkinson’s Disease
Mohamad Ayajuddin1†, Rahul Chaurasia1†, Abhik Das1†, Priyanka Modi1†, Limamanen Phom1,2, Zevelou Koza1,3, and Sarat Chandra Yenisetti1*

## Slide 2
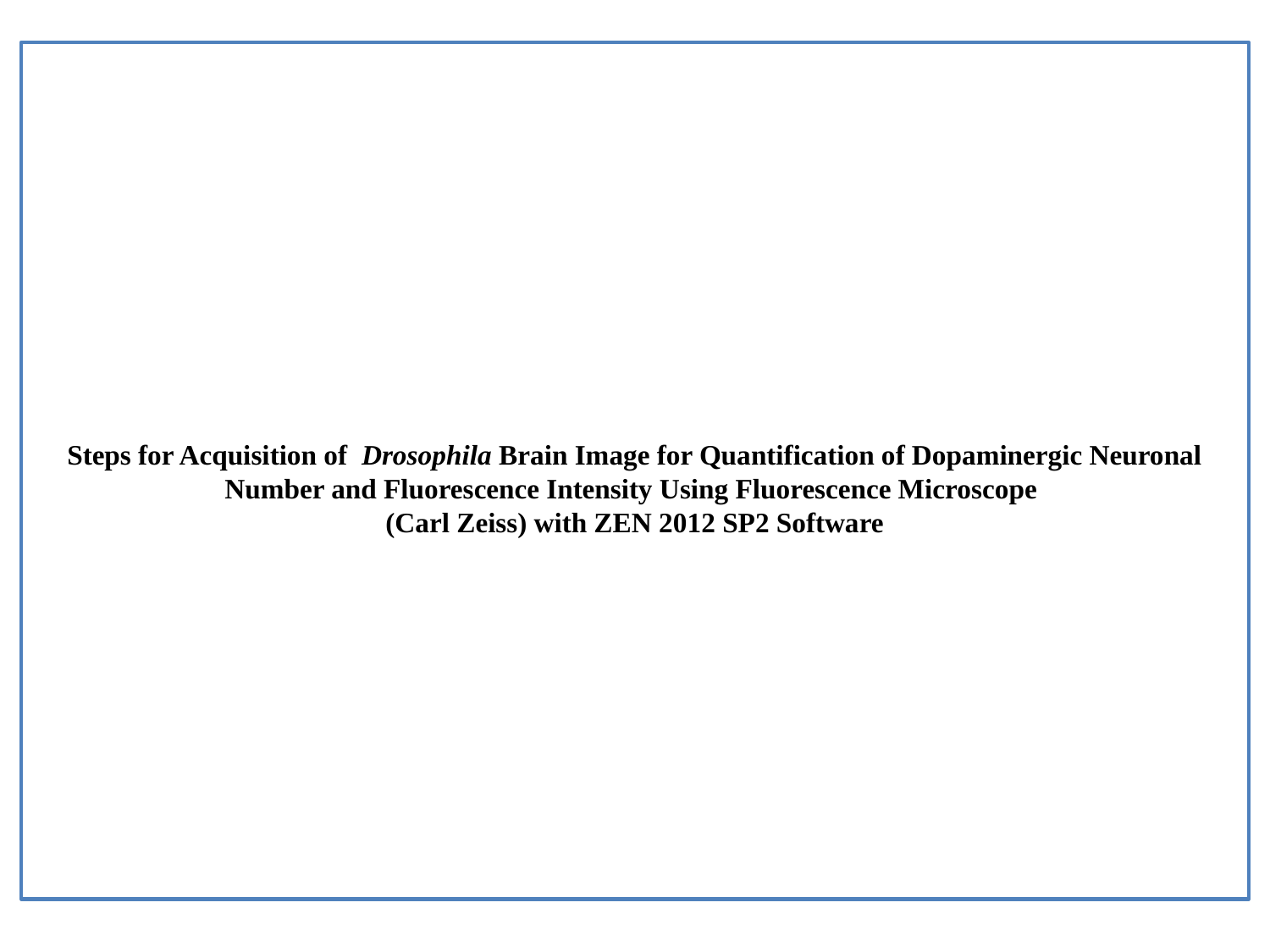

# Steps for Acquisition of Drosophila Brain Image for Quantification of Dopaminergic Neuronal Number and Fluorescence Intensity Using Fluorescence Microscope (Carl Zeiss) with ZEN 2012 SP2 Software

## Slide 3
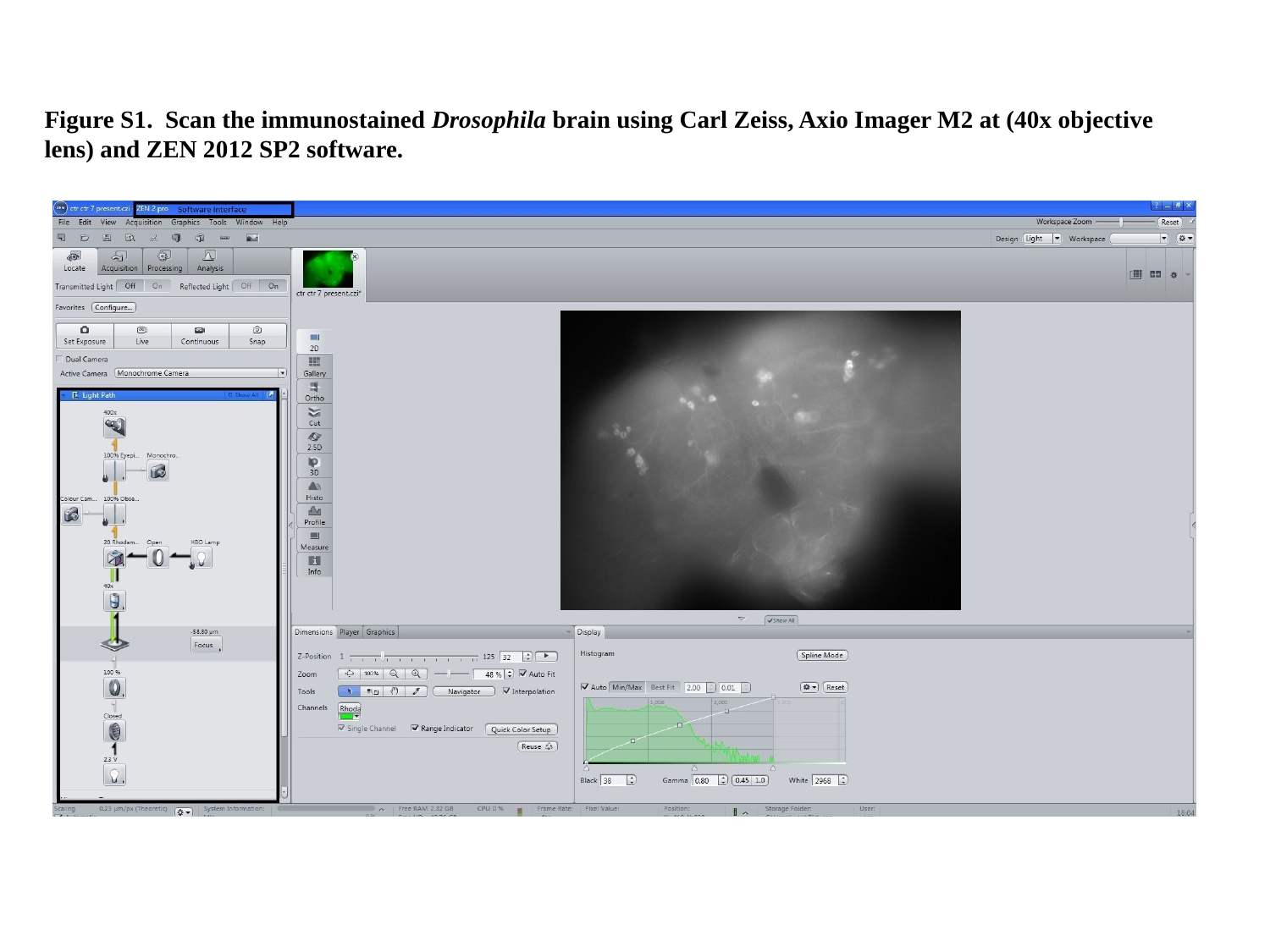

Figure S1. Scan the immunostained Drosophila brain using Carl Zeiss, Axio Imager M2 at (40x objective lens) and ZEN 2012 SP2 software.

## Slide 4
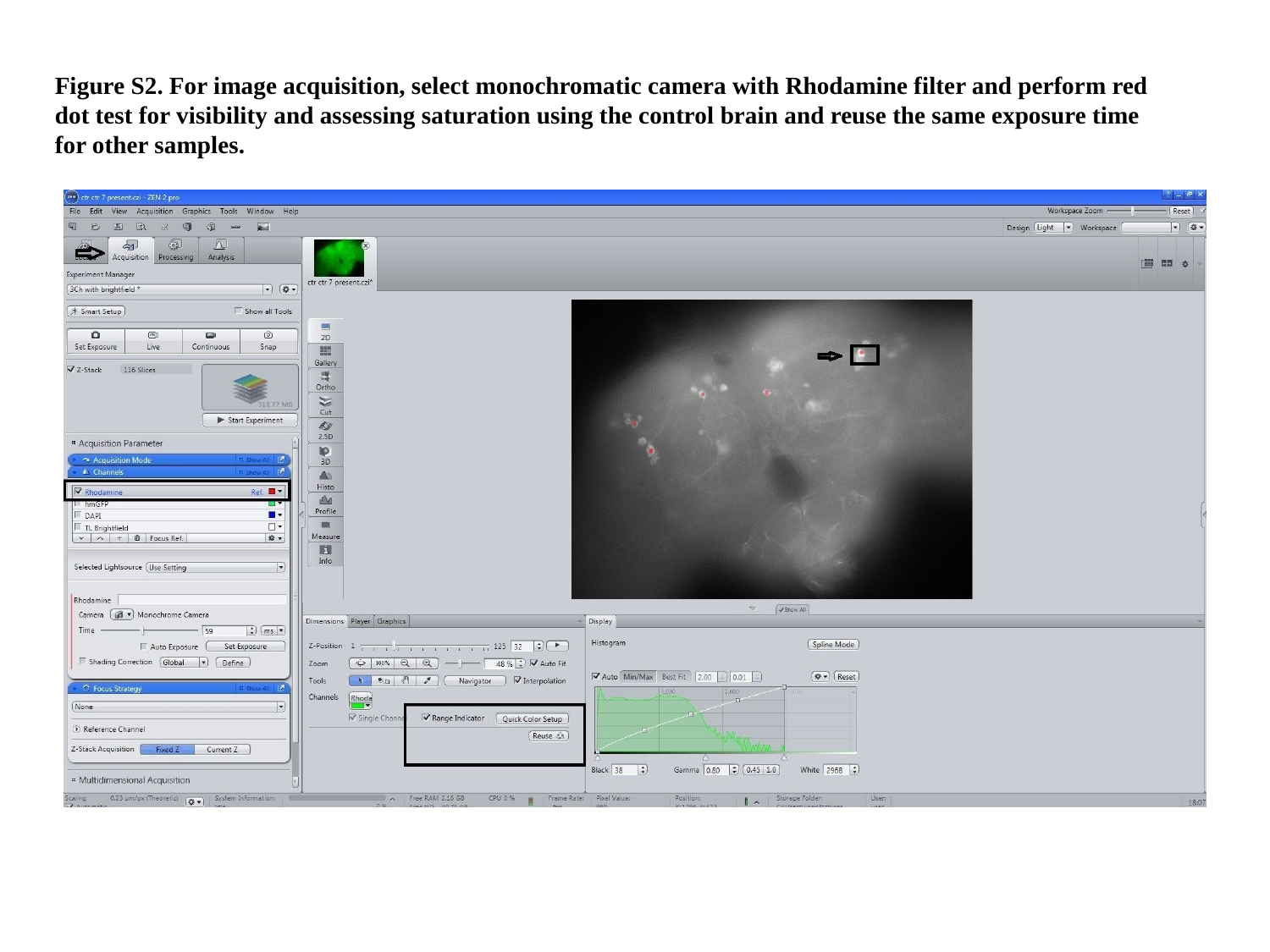

Figure S2. For image acquisition, select monochromatic camera with Rhodamine filter and perform red dot test for visibility and assessing saturation using the control brain and reuse the same exposure time for other samples.

## Slide 5
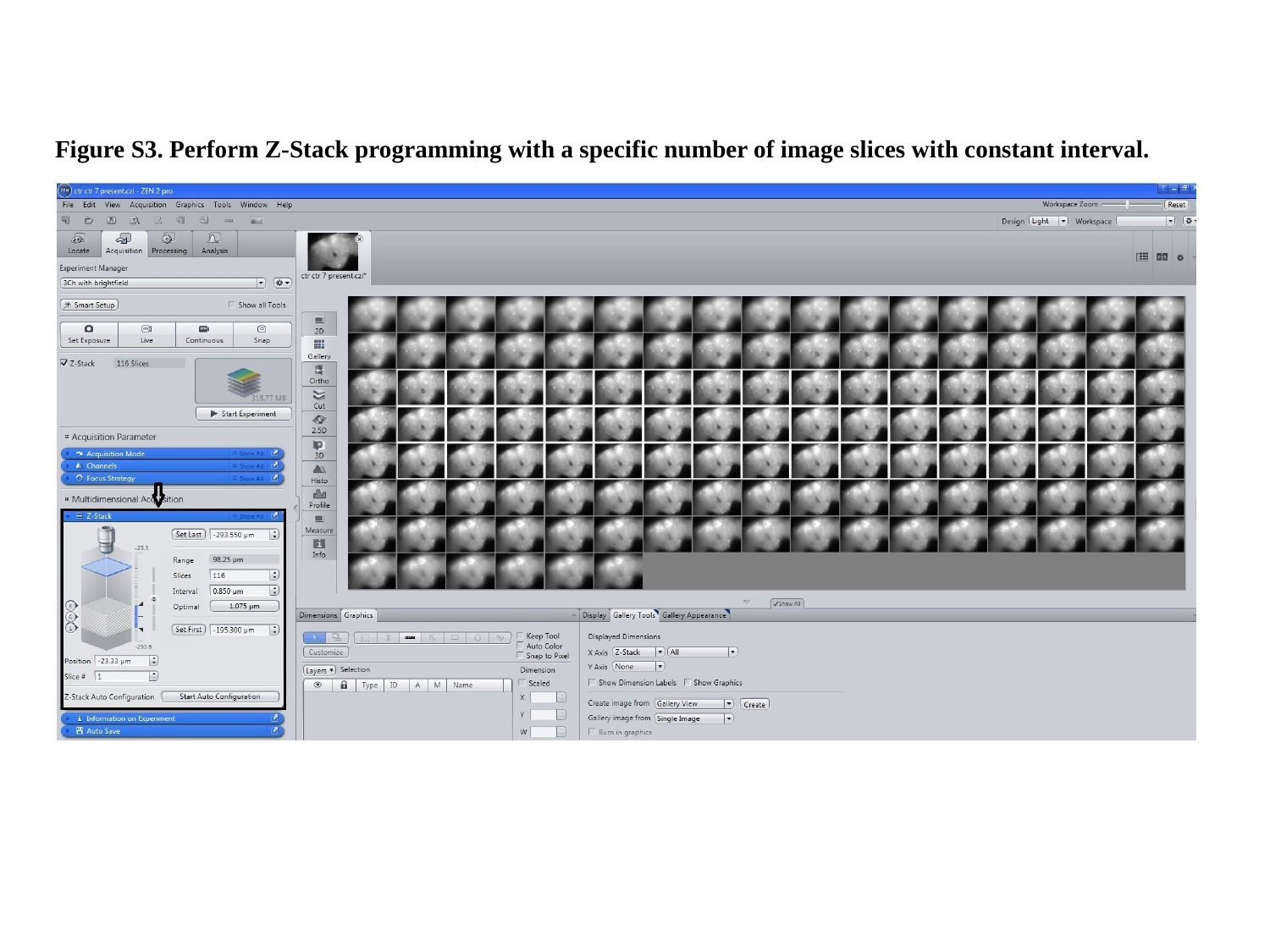

Figure S3. Perform Z-Stack programming with a specific number of image slices with constant interval.

## Slide 6
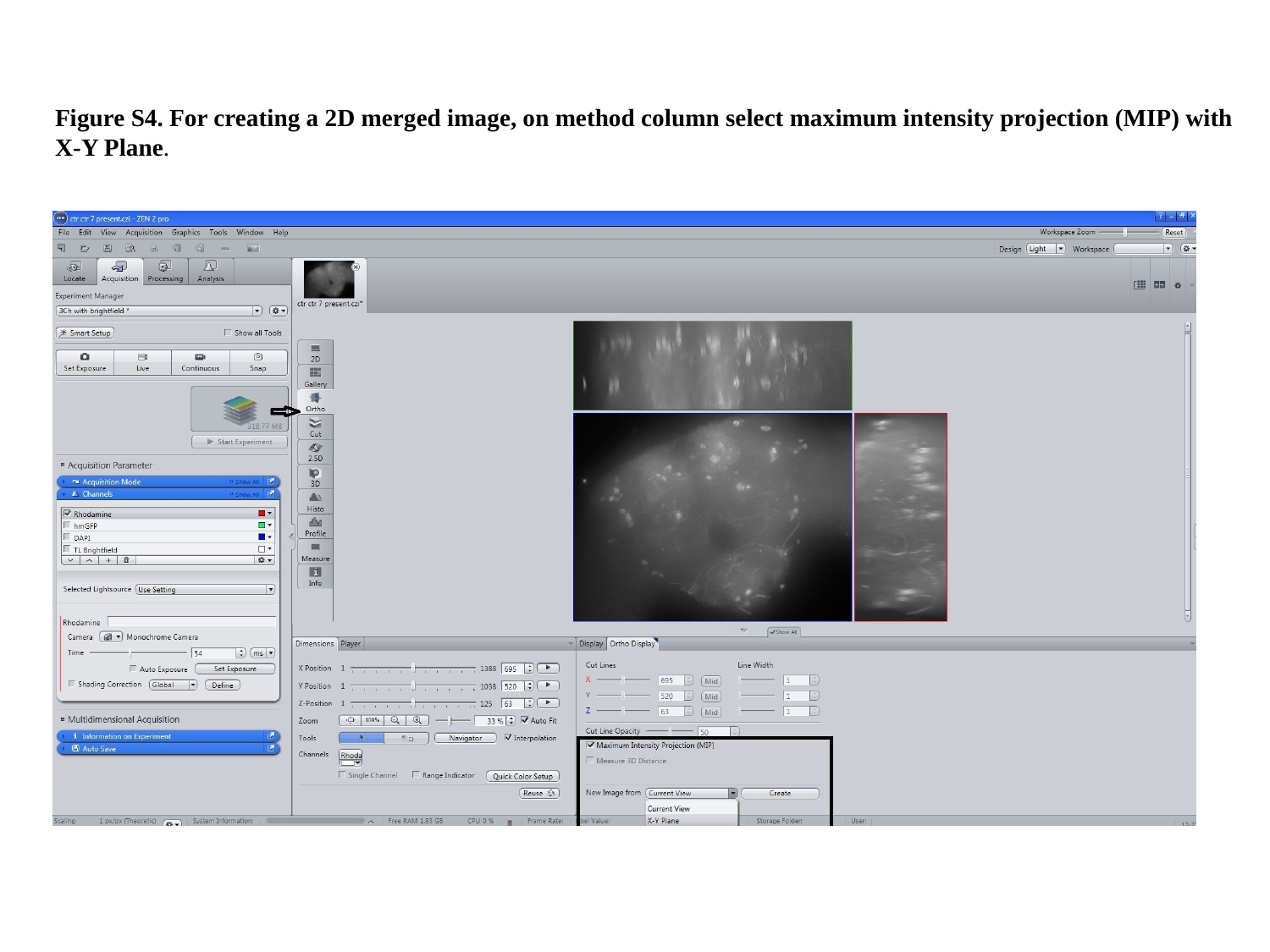

Figure S4. For creating a 2D merged image, on method column select maximum intensity projection (MIP) with X-Y Plane.

## Slide 7
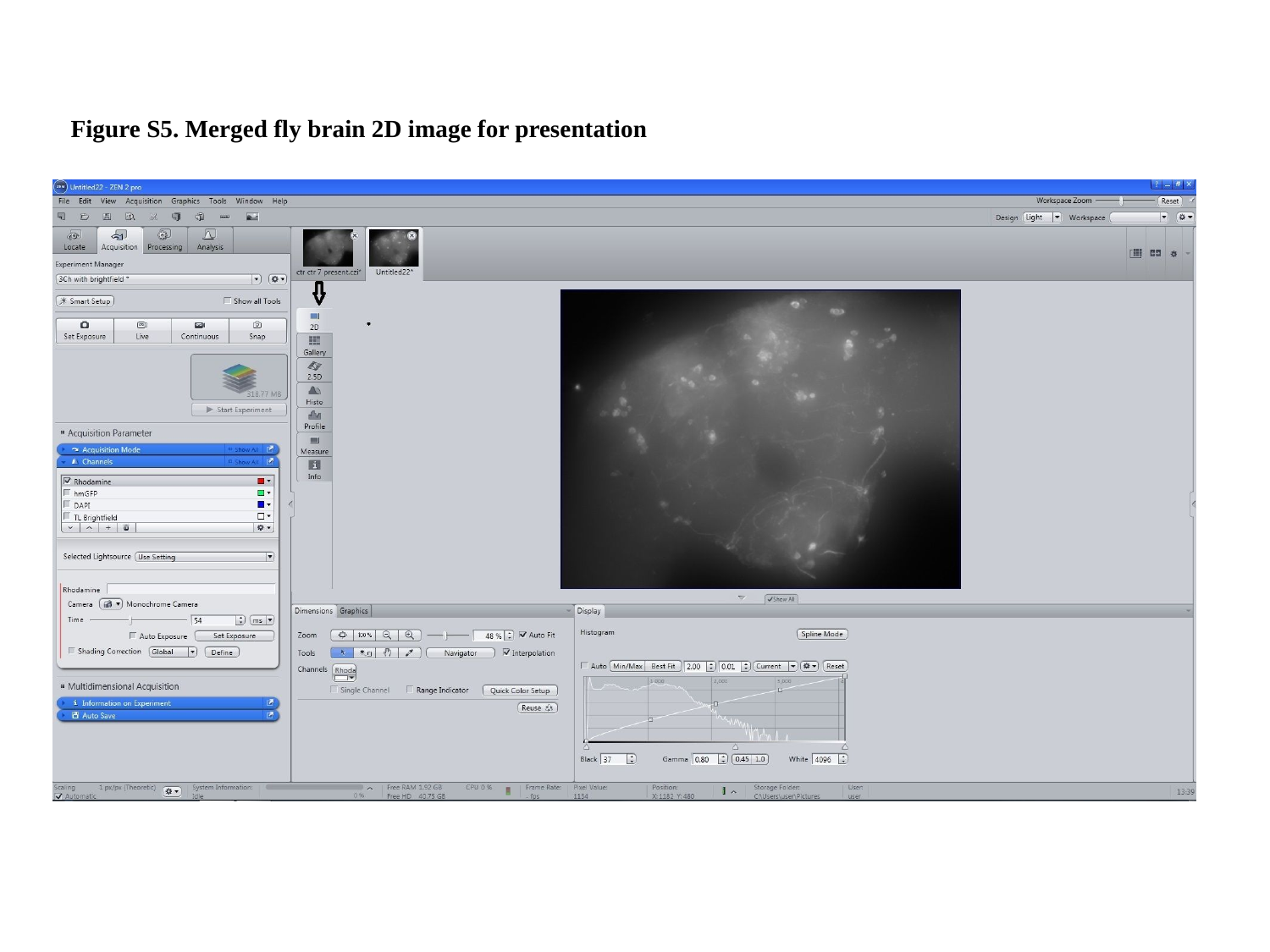

Figure S5. Merged fly brain 2D image for presentation

## Slide 8
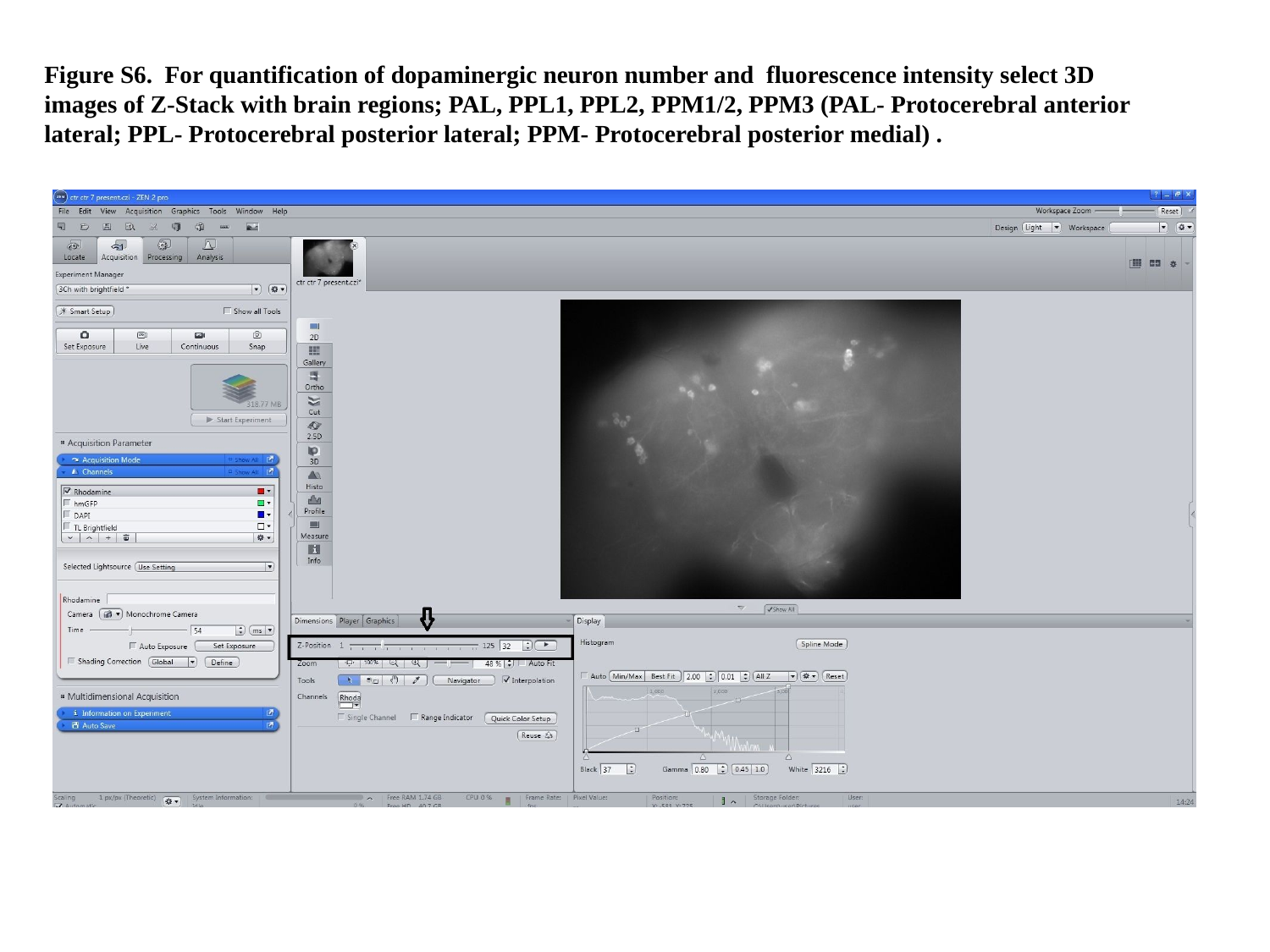

Figure S6. For quantification of dopaminergic neuron number and fluorescence intensity select 3D images of Z-Stack with brain regions; PAL, PPL1, PPL2, PPM1/2, PPM3 (PAL- Protocerebral anterior lateral; PPL- Protocerebral posterior lateral; PPM- Protocerebral posterior medial) .

## Slide 9
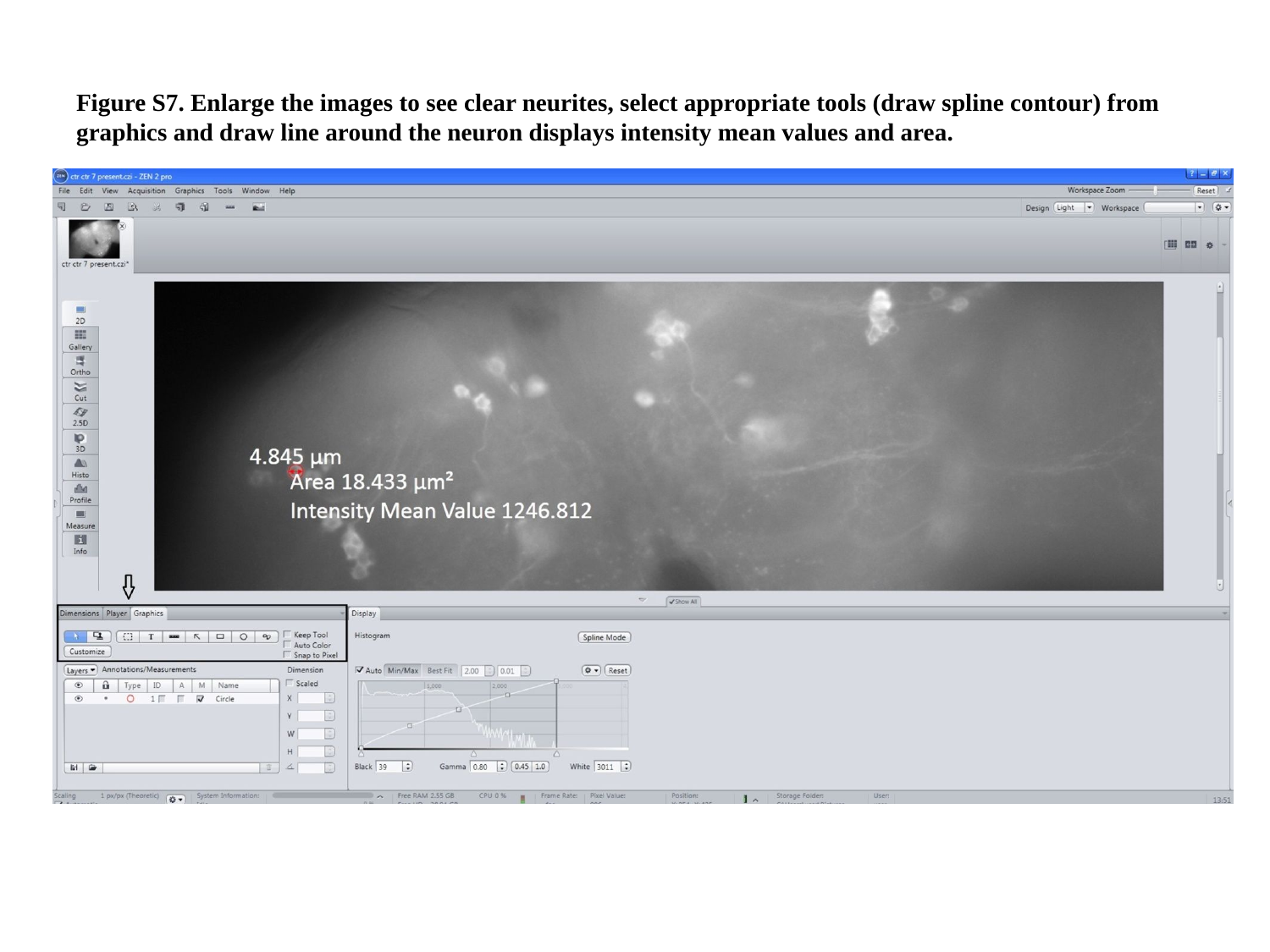

Figure S7. Enlarge the images to see clear neurites, select appropriate tools (draw spline contour) from graphics and draw line around the neuron displays intensity mean values and area.

## Slide 10
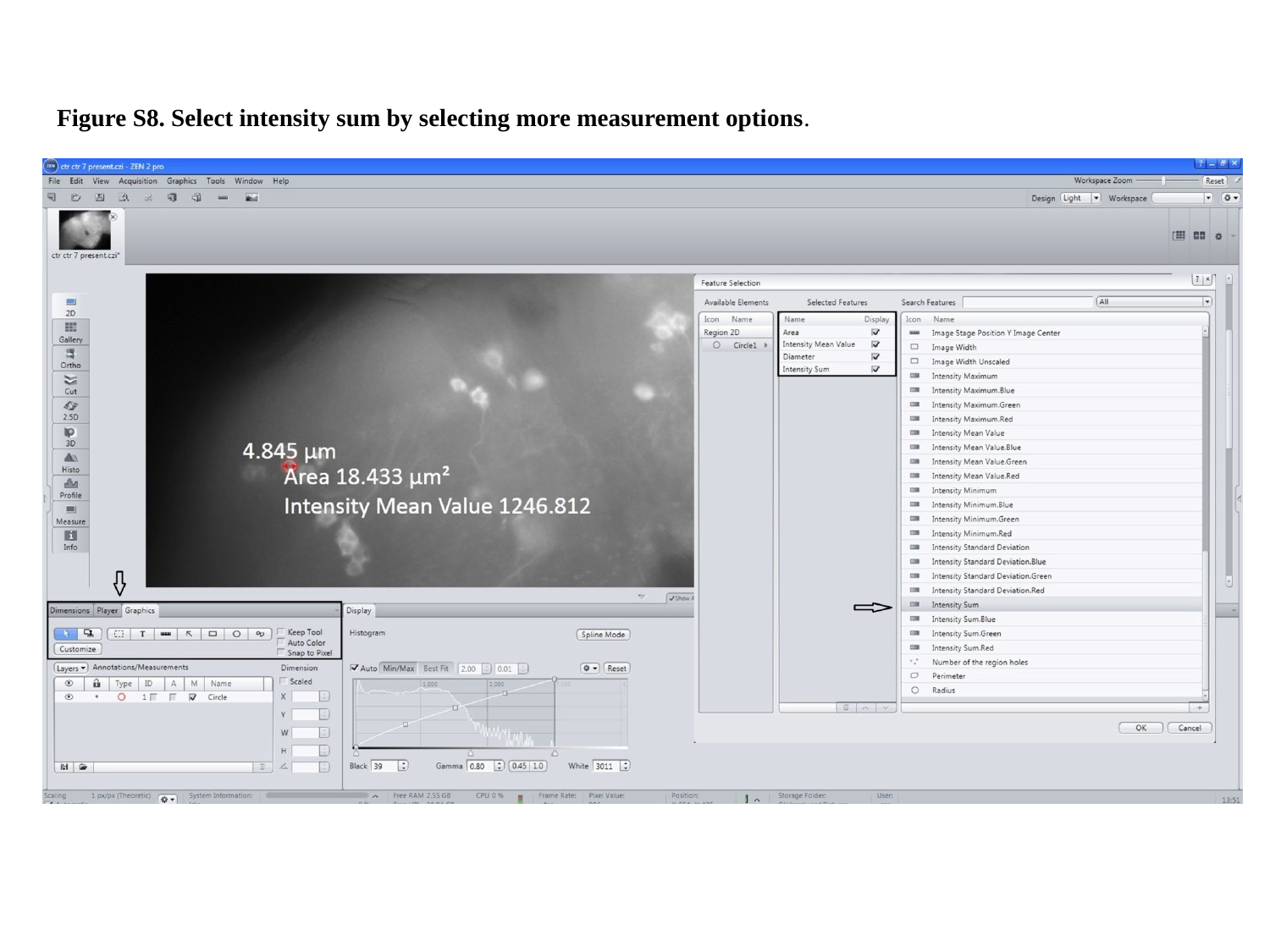

Figure S8. Select intensity sum by selecting more measurement options.

## Slide 11
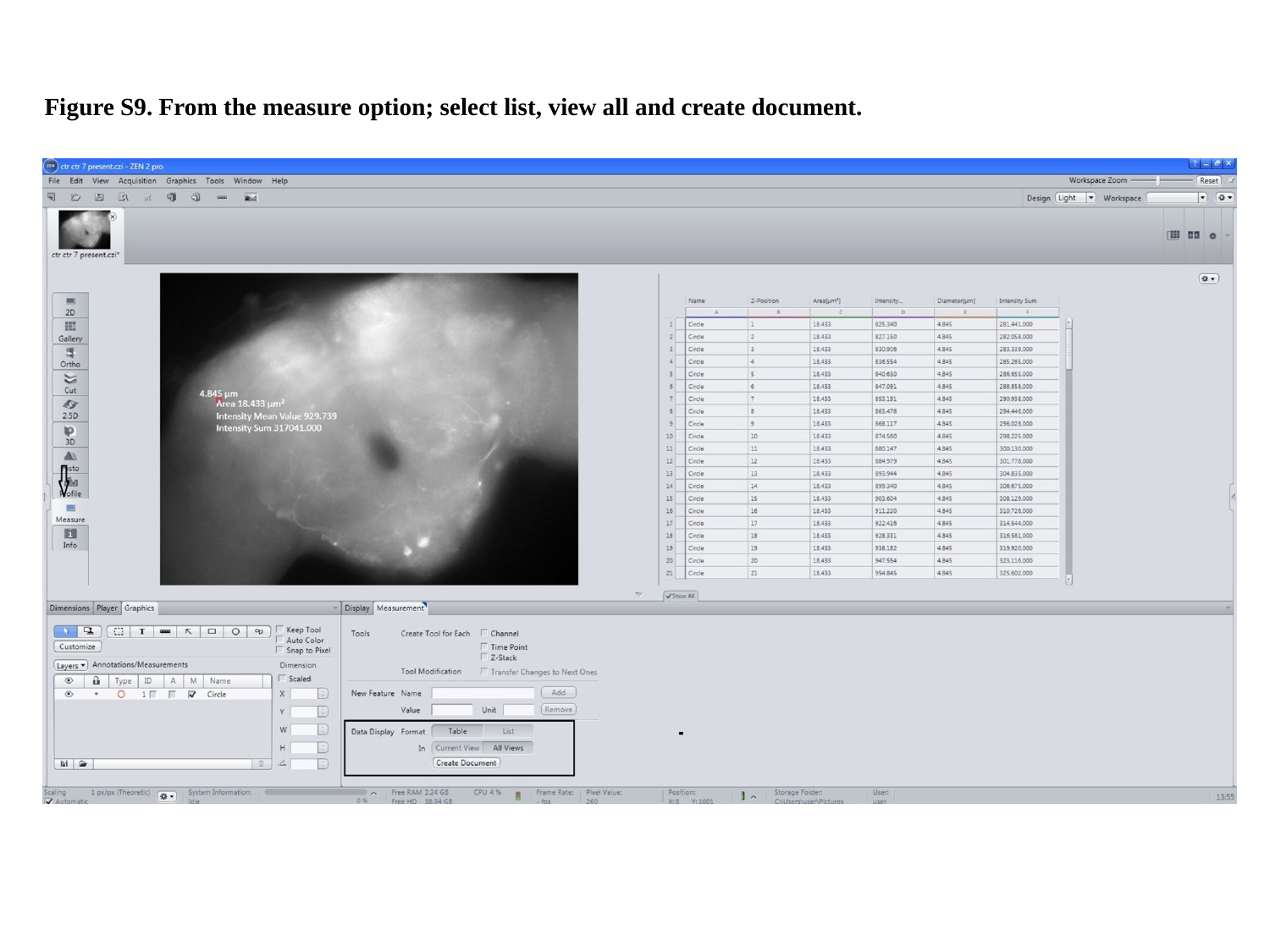

Figure S9. From the measure option; select list, view all and create document.

## Slide 12
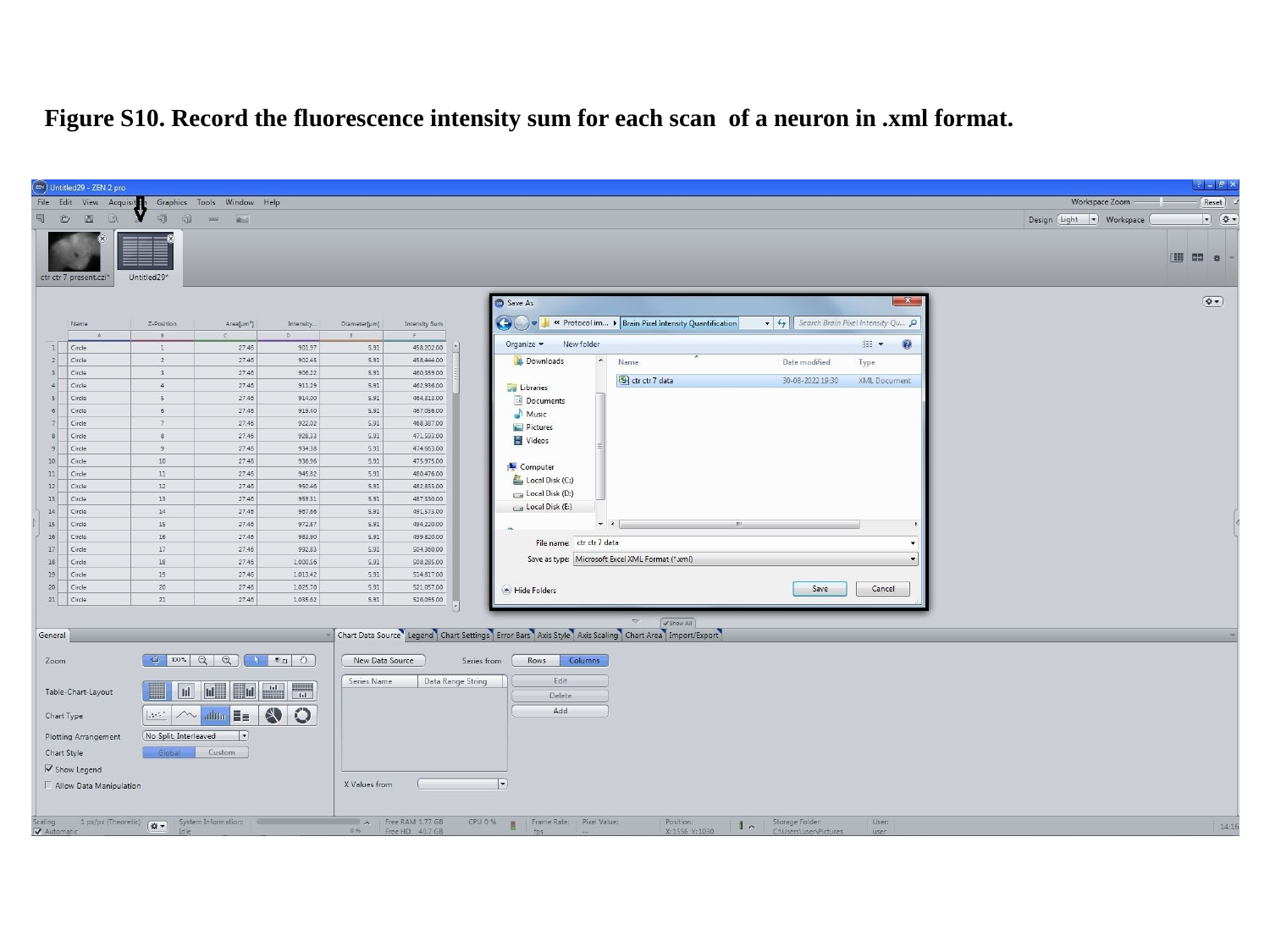

Figure S10. Record the fluorescence intensity sum for each scan of a neuron in .xml format.

## Slide 13
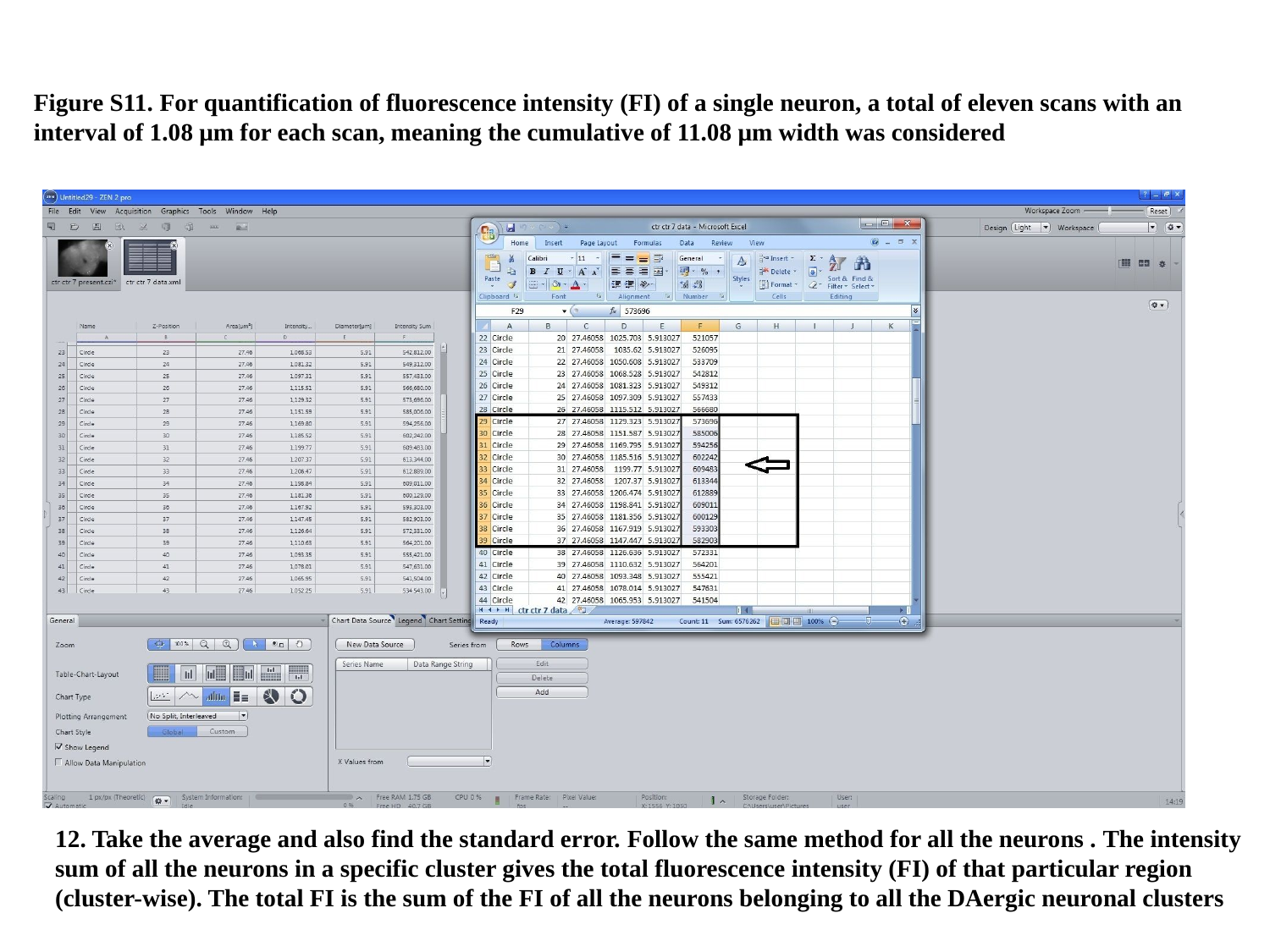

Figure S11. For quantification of fluorescence intensity (FI) of a single neuron, a total of eleven scans with an interval of 1.08 μm for each scan, meaning the cumulative of 11.08 μm width was considered
12. Take the average and also find the standard error. Follow the same method for all the neurons . The intensity sum of all the neurons in a specific cluster gives the total fluorescence intensity (FI) of that particular region (cluster-wise). The total FI is the sum of the FI of all the neurons belonging to all the DAergic neuronal clusters
